# Supplementary material for: Self-assembly of metal–organic polyhedra into supramolecular polymers with intrinsic microporosity
Source: Nat Commun. 2018 Jul 12;9:2506. doi: 10.1038/s41467-018-04834-0 (PMC6043503; doi:10.1038/s41467-018-04834-0)
Supplement: Supplementary file 1 — Supplementary Information [file 41467_2018_4834_MOESM1_ESM.pdf]

Supplementary Information

Self-assembly of metal-organic polyhedra into supramolecular polymers with intrinsic microporosity

Arnau Carné-Sánchez *et al*.

Supplementary Figures

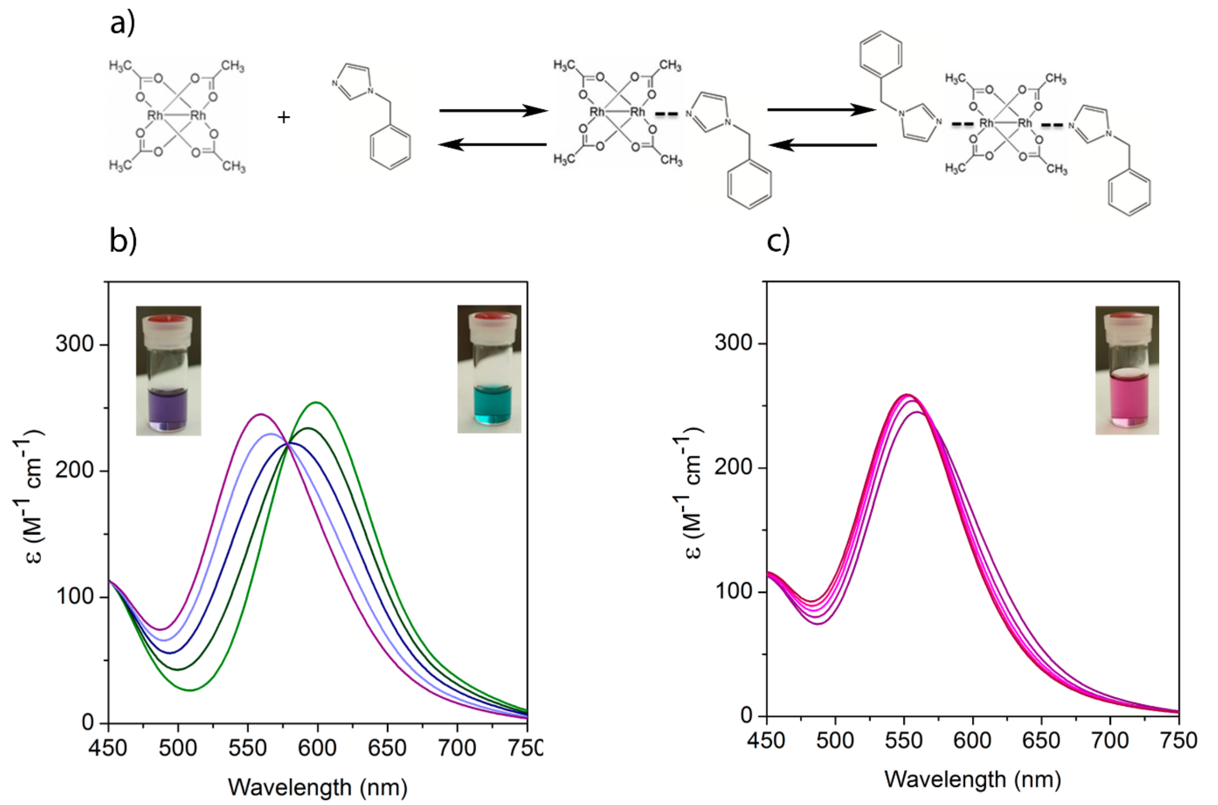

Supplementary Figure 1. (a) Reaction scheme of $[Rh_2(OAc)_4]$ with biz. (b) UV-Vis spectra of the titration of $[Rh_2(OAc)_4]$ (DMF solution, 4.96 mM) with biz from 0 mol. eq. (green) to 1 mol. eq. (purple). (Inset) Photographs show the initial colour of the $[Rh_2(OAc)_4]$ solution (right) and after the addition of 1 eqv of biz (left). (c) UV-Vis spectra of the titration of $[Rh_2(OAc)_4]$ from 1 mol. eq. of biz to 7 mol. eq.. (Inset) Photograph showing a solution of $[Rh_2(OAc)_4]$ in the presence of 7 mol. eq. of biz.

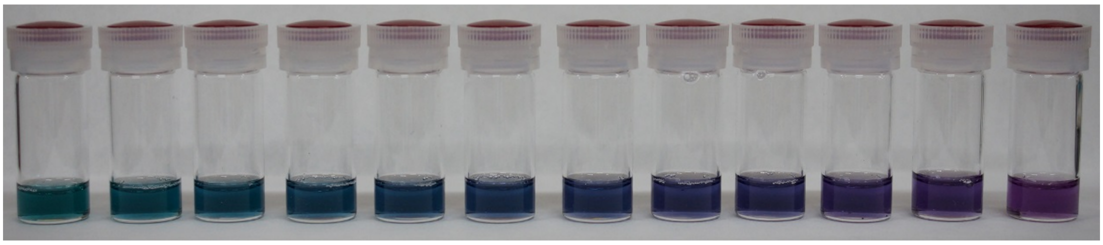

Supplementary Figure 2. Photograph of a DMF solution of C_{12}RhMOP (0.23 mM) reacted with increasing molar equivalents (mol. eq.) of biz: 0 to 12 mol. eq. (from left to right).

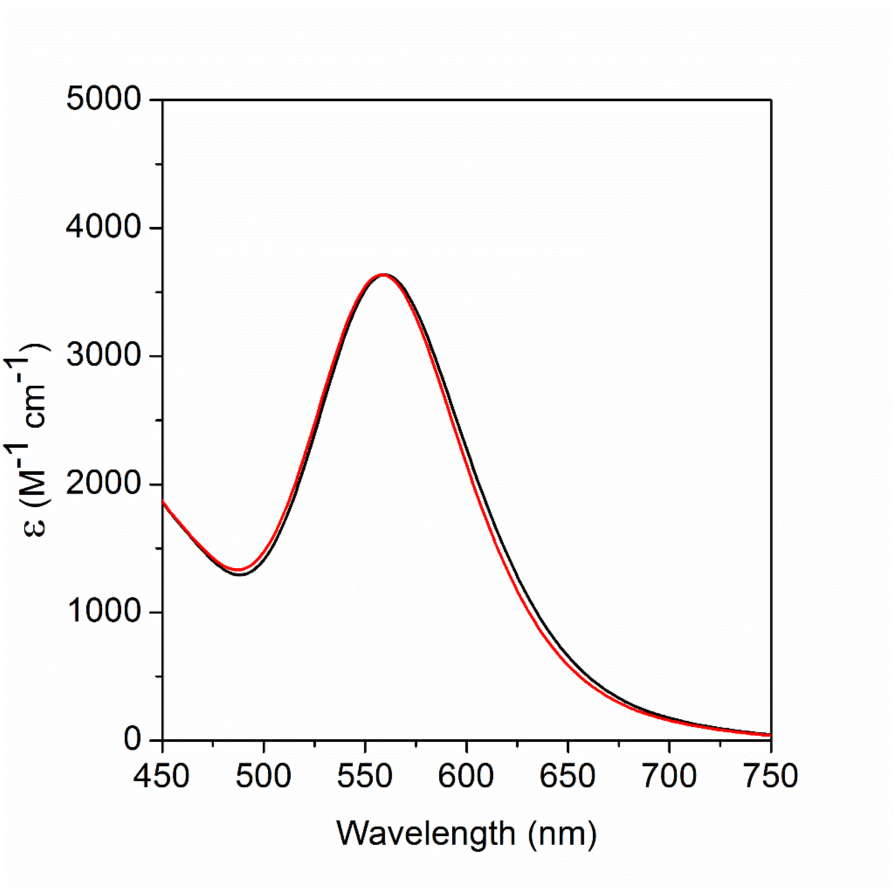

Supplementary Figure 3. UV-Vis spectra of C_{12}RhMOP (0.23 mM) after addition of 12 mol. eq. (black) and 24 mol. eq. (red) of biz.

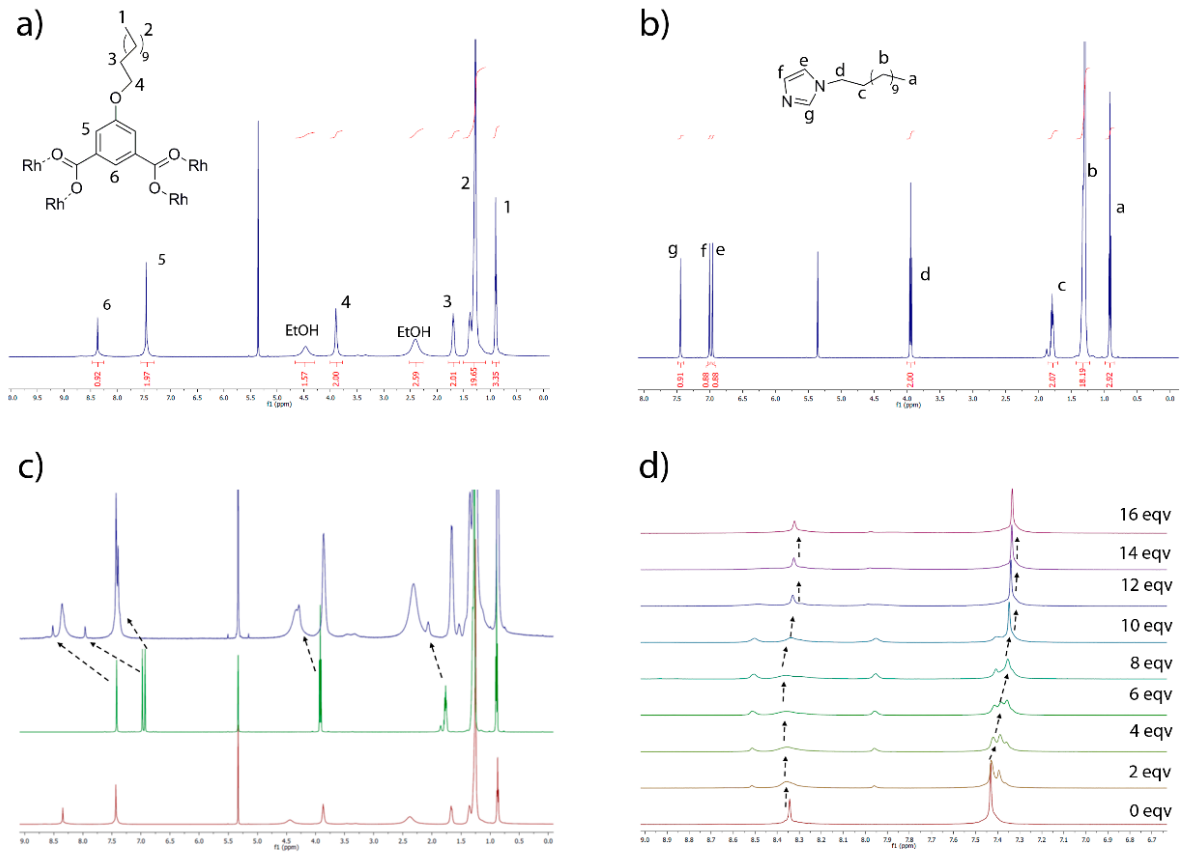

Supplementary Figure 4. a) ^{1}H-NMR spectrum of C_{12}RhMOP in DCM-d_2. b) ^{1}H-NMR spectrum of dis in DMSO-d^6. c) stacked ^{1}H-NMR spectra of C_{12}RhMOP (red), dis (green) and C_{12}RhMOP in the presence of 2 eqv of dis (blue) in DCM-d_2. Arrows highlight the shift of dis chemical shifts when coordinated to the Rh-Rh paddle wheel. d) stacked NMR spectra of the titration of C_{12}RhMOP with dis. Arrows highlight the chemical shift of the aromatic protons of the MOP. Note that the extent of shift is greater for the protons pointing outside the MOP cage (proton 5 shifts from 7.46 ppm to 7.36 ppm) when compared to the proton pointing inside (proton 6 shifts from 8.37 ppm to 8.35 ppm) and that there is no further shift after the addition of 12 mol. eq. of dis. These results further support the hypothesis that coordination only occurs on the exohedral axial sites of the C_{12}RhMOP. Note that the NMR experiments were performed with an aliphatic imidazole rather than the aromatic biz in order to reduce the number of signals at the aromatic region.

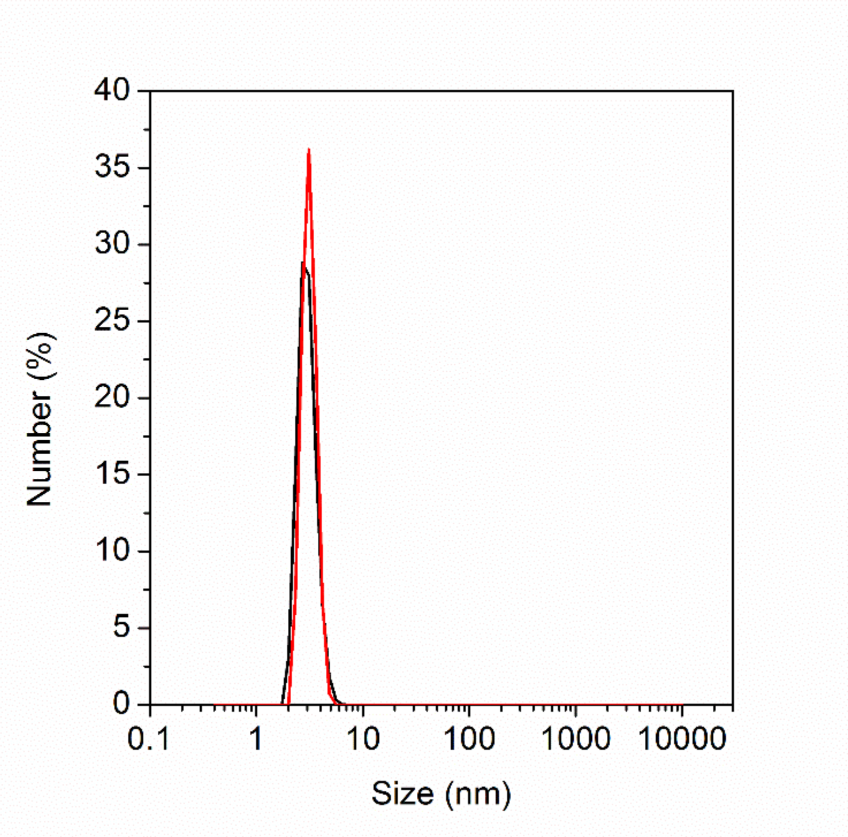

Supplementary Figure 5. DLS measurement of C_{12}RhMOP (black) and C_{12}RhMOP after addition of 12 mol. eq. of biz.

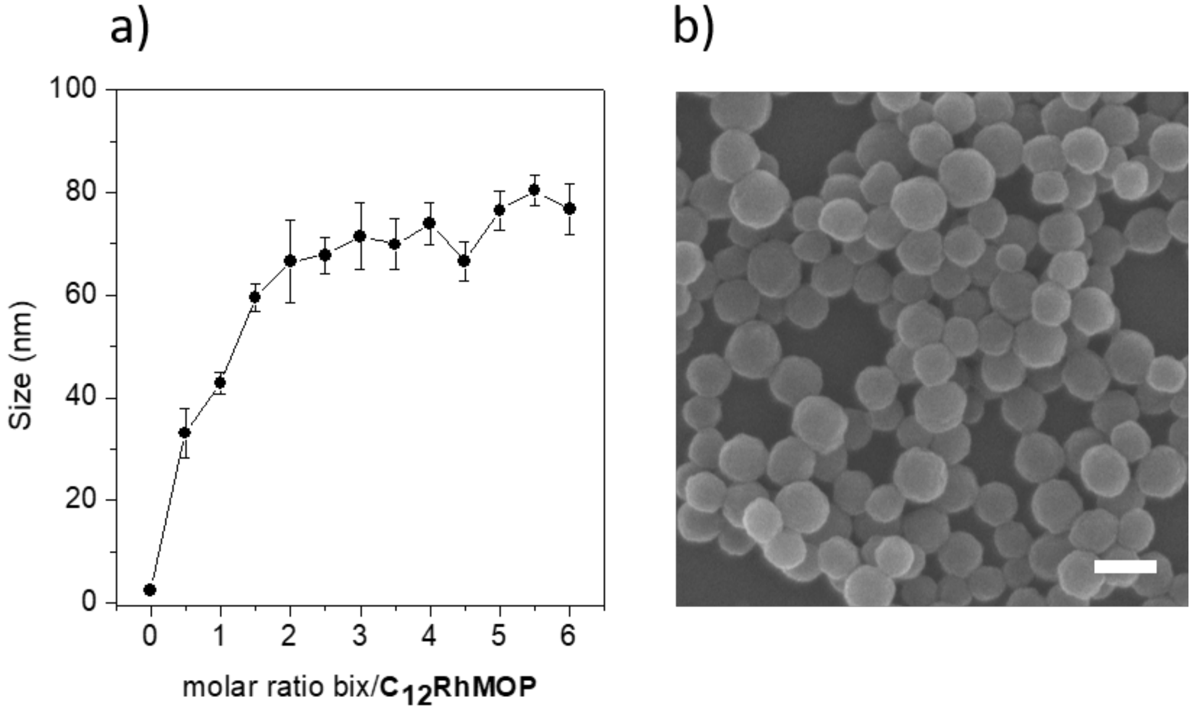

Supplementary Figure 6. (a) DLS titration of C_{12}RhMOP (0.23 mM). (b) FESEM image of the coordination polymer particles obtained after titration. Scale bar: 100 nm

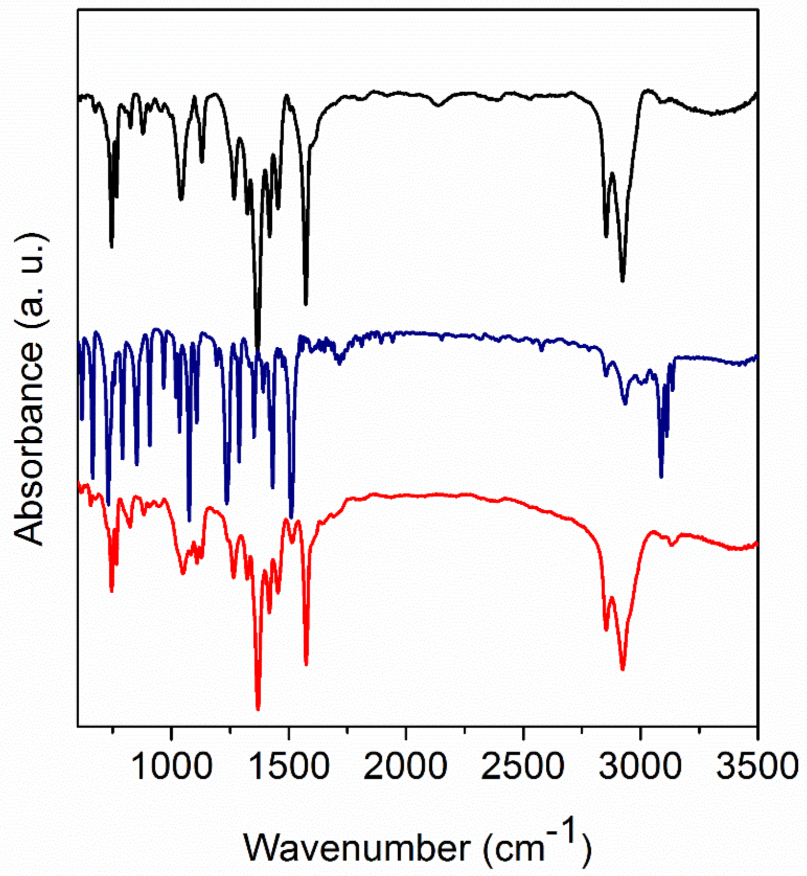

Supplementary Figure 7. FT-IR spectra of C_{12}RhMOP (black), bix (blue) and CPP-1 (red).

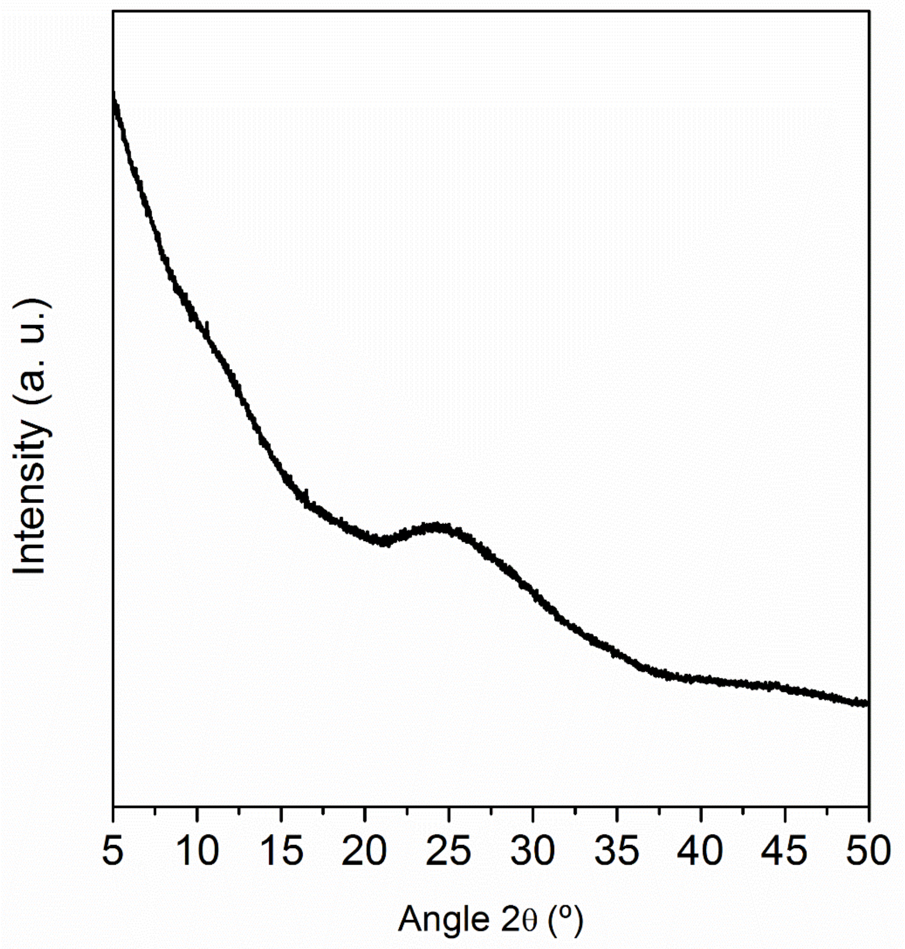

Supplementary Figure 8. Powder X-ray diffractogram of CPP-1. The broad peaks arise from the glass substrate.

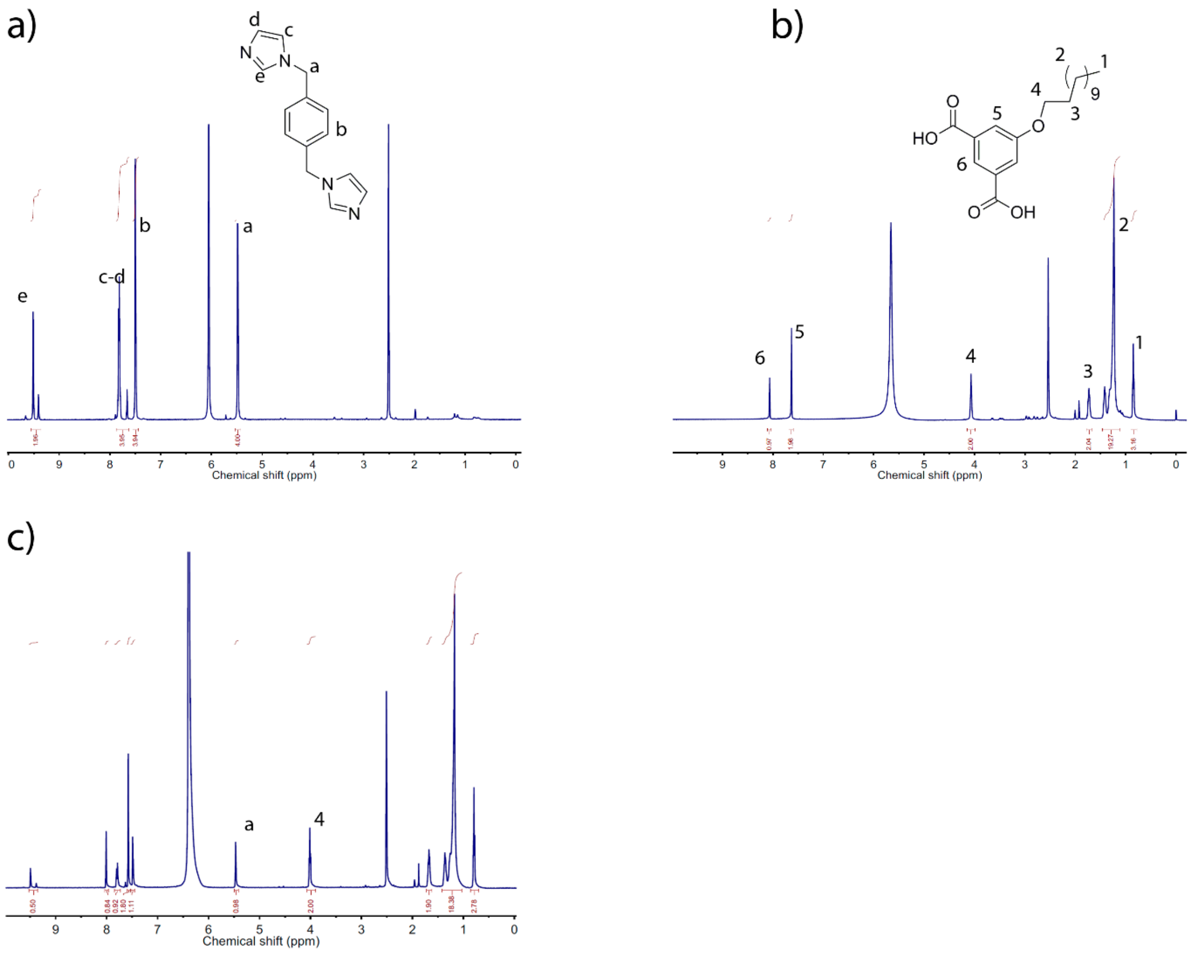

Supplementary Figure 9. (a) ^{1}H-NMR spectrum of bix treated with the same conditions used to digest the C_{12}RhMOP and the coordination polymers derived from it. (b) ^{1}H-NMR spectrum of digested C_{12}RhMOP. (c) ^{1}H-NMR spectrum of digested CPP-1. The ratio of the integrals of proton 4 from the MOP and proton "a" from bix was used to determine the molar ratio between the two ligands. Thus, in the case of CPP-1 the ratio of bdc-C_{12}:bix = 1:0.25 which corresponds to the following formula: C_{12}RhMOP(bix)$_6$.

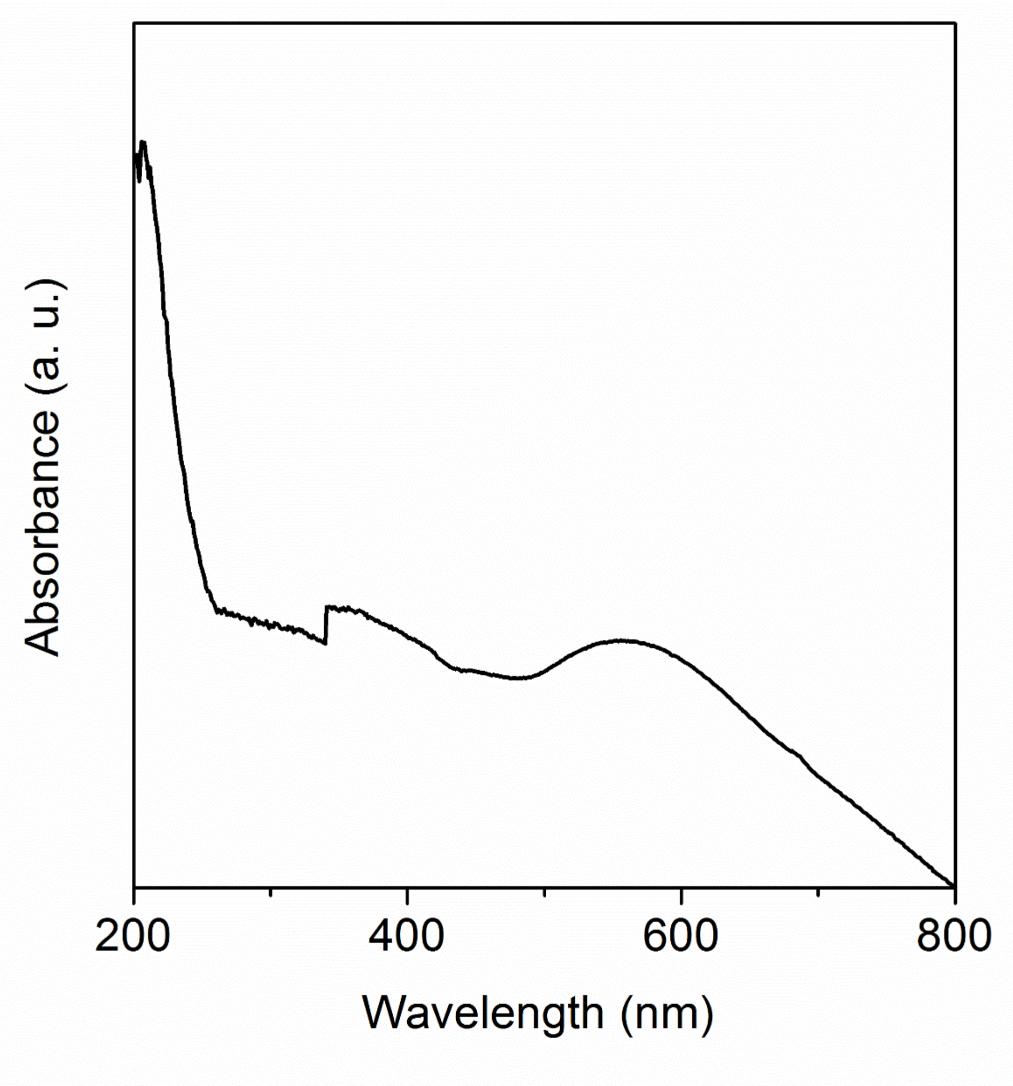

Supplementary Figure 10. Solid state UV-Vis spectrum of CPP-1, λ_{max} = 560 nm.

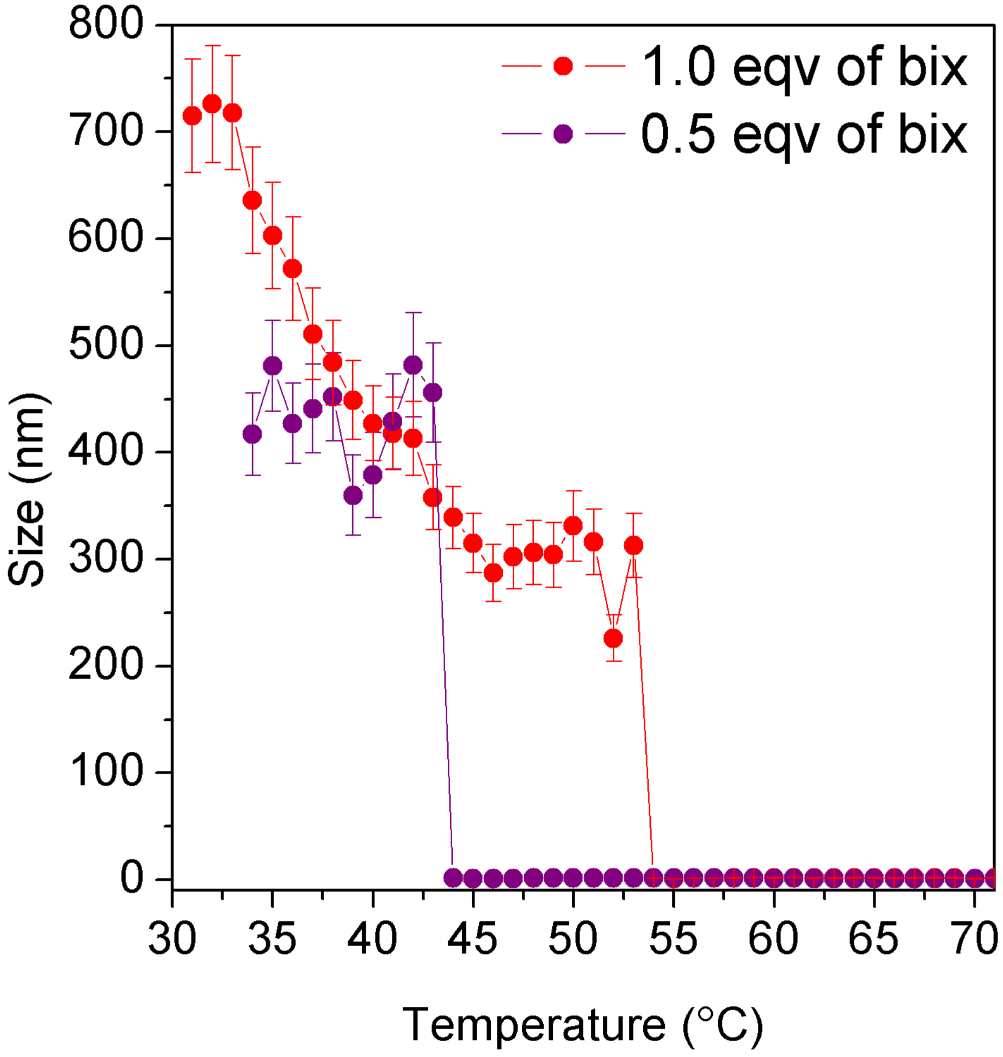

Supplementary Figure 11. DLS monitoring of the size evolution of CPP-1 nuclei (0.93 mM) synthesised after adding 1 eqv of bix (red) or 0.5 eqv of bix (purple). The addition of 1.5 eqv of bix at 70 ºC resulted in immediate precipitation at this concentration.

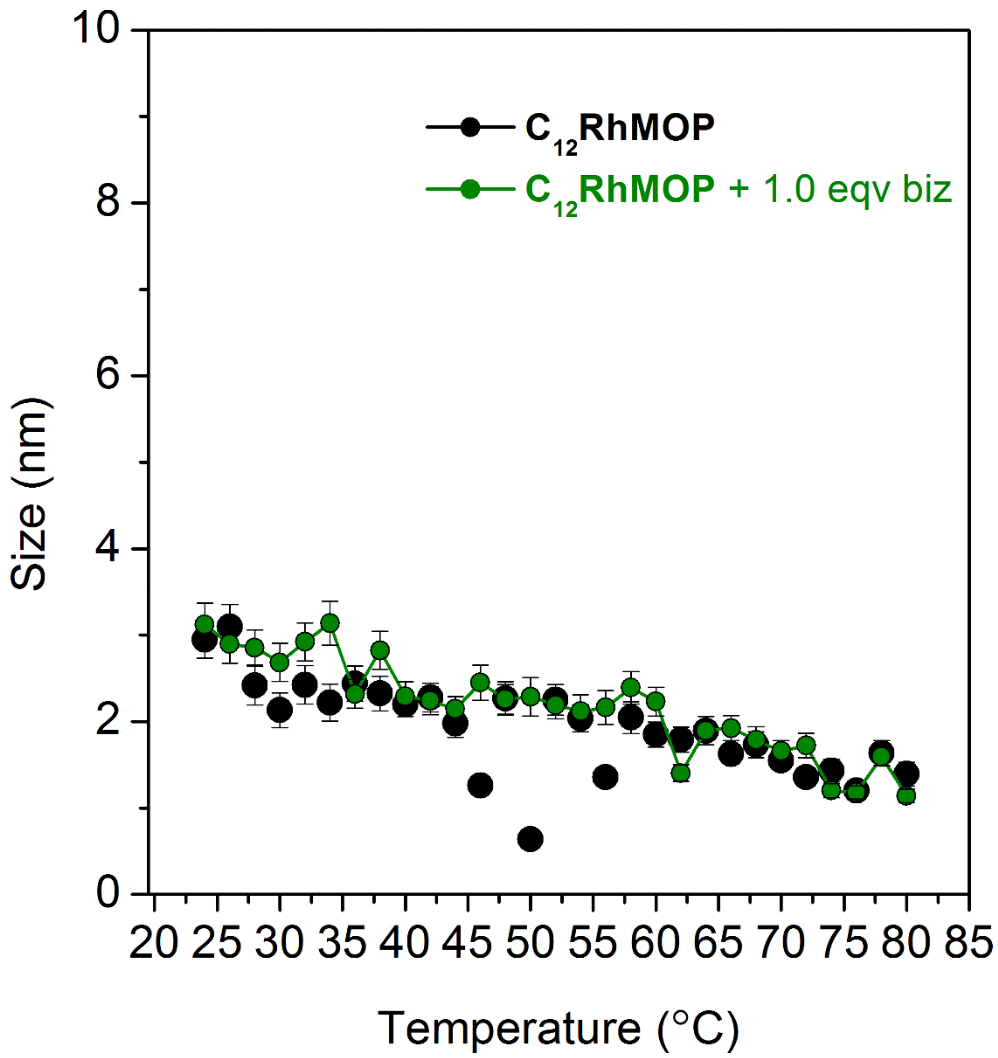

Supplementary Figure 12. Temperature variable DLS experiments performed on C_{12}RhMOP (0.93 mM) and C_{12}RhMOP in the presence of 1 mol. eq. of biz.

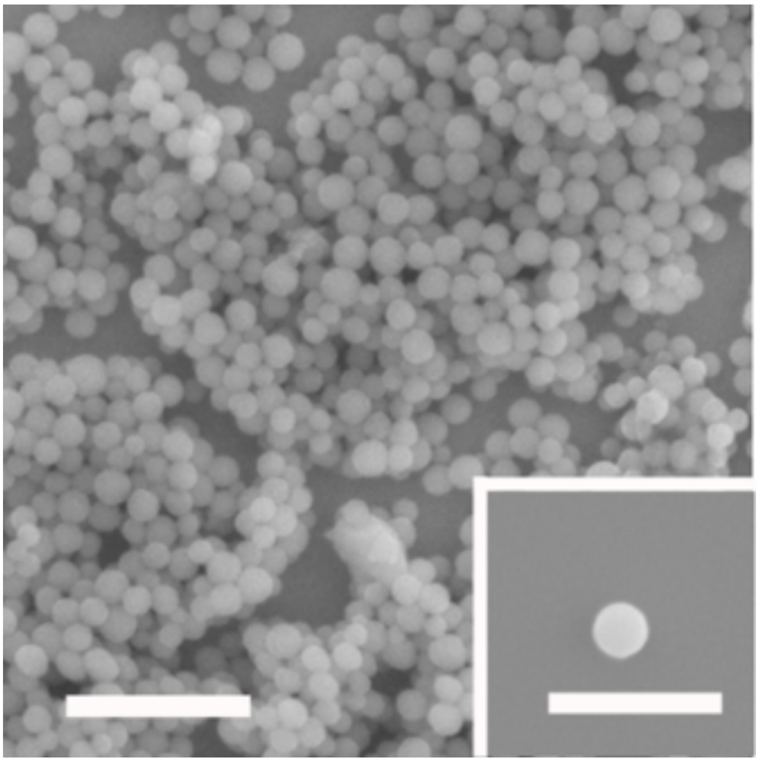

Supplementary Figure 13. Representative FESEM image of the CPP-1 synthesized at a C_{12}RhMOP concentration of 0.93 mM and 0.5 mol. eq. of bix per step. Scale bar: µm (inset: 500 nm).

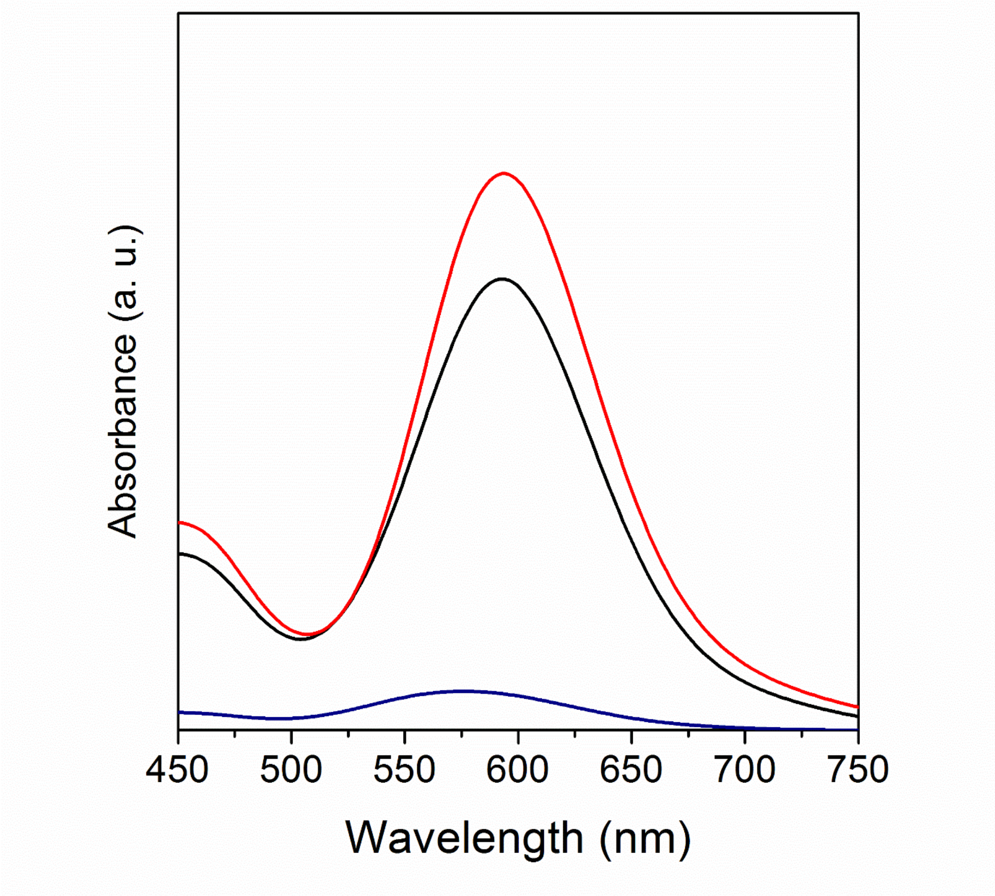

Supplementary Figure 14. UV-vis spectra of the initial C_{12}RhMOP solution (λ_{max} = 593 nm, red), the supernatant obtained after adding 1 mol. eq. of bix (λ_{max} = 593 nm, black), and 4 mol. eq. of bix (λ_{max} = 576 nm, blue). The fact that addition of 1 mol. eq. of bix does not induce a shift in λ_{max} further confirms that at the initial stage of the titration all of the bix molecules are employed in generating nuclei, leaving uncoordinated C_{12}RhMOP in solution. However, after adding 4 mol. eq. of bix, C_{12}RhMOP is consumed and only a small fraction of uncoordinated species remain in solution. Because the final composition of CPP-1 has 6 mol. eq. of bix, it is assumed that after the growth process the particles formed *in situ* increase their coordination number by incorporating bix.

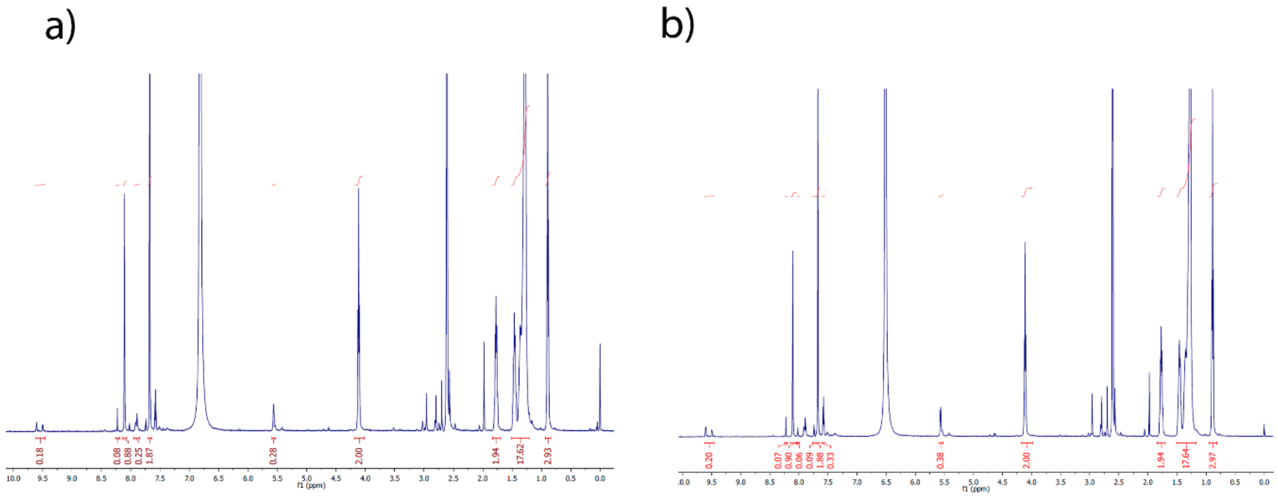

Supplementary Figure 15. ^{1}H-NMR spectra for digestions of the CPP-1 nuclei obtained after adding (a) 1 mol. eq. and (b) 1.5 mol. eq. of bix. The ratio of bdc-C_{12}:Bix was 0.07 for the case of CPP-1 and 0.095 in the case of the 1.5 seeds. Thus the formulae were $C_{12}RhMOP(bix)_{1.7}$ and $C_{12}RhMOP(bix)_{2.3}$, respectively.

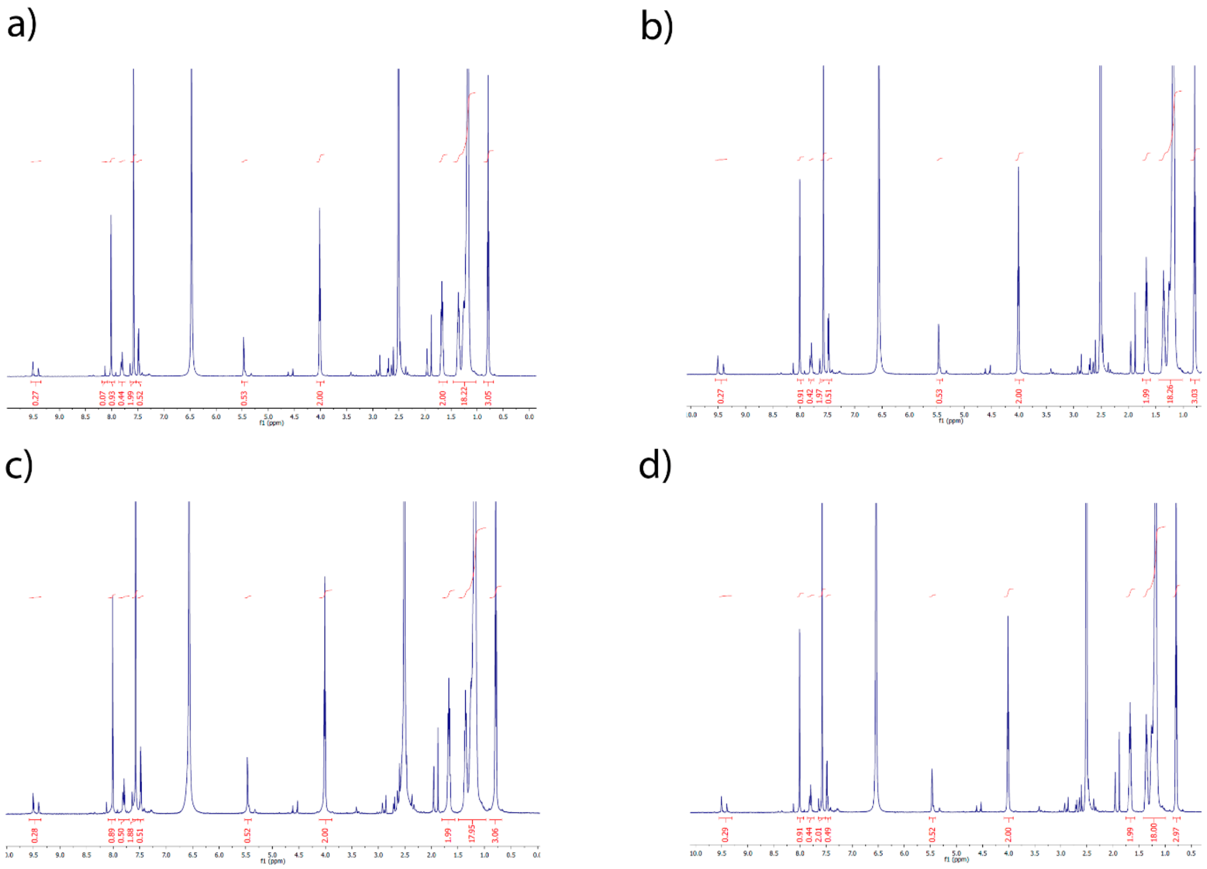

Supplementary Figure 16. ^{1}H-NMR of the nuclei obtained after adding a total of 2 mol. eq. of bix using different step sizes: (a) 0.25 mol. eq., (b) 0.5 mol. eq., (c) 1 mol. eq., and (d) 2 mol. eq.. In all cases the ratio of bdc-C_{12}: bix was 0.13 and therefore the formula was C_{12}RhMOP(bix)$_3$.

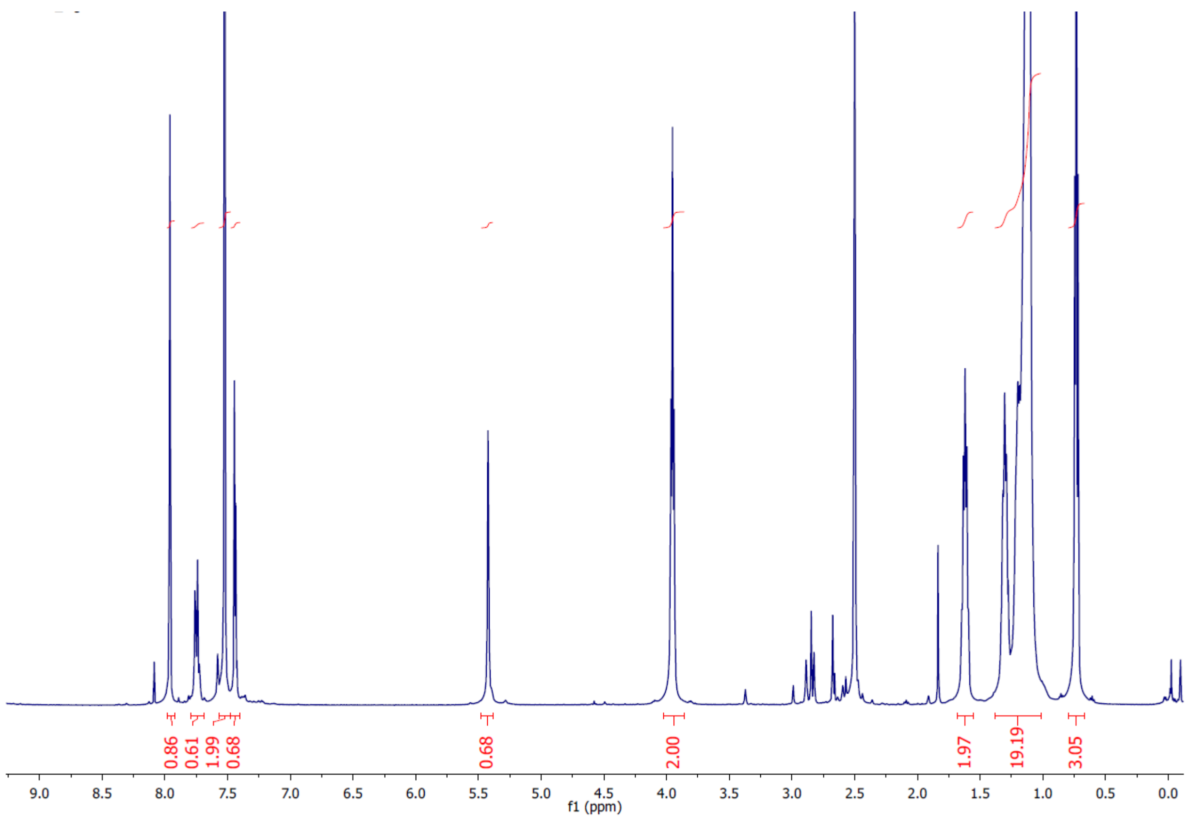

Supplementary Figure 17. ^{1}H-NMR spectrum of digested CPP-1 particles after adding 4 mol. eq. of bix. The corresponding formula was C_{12}RhMOP(bix)$_4$.

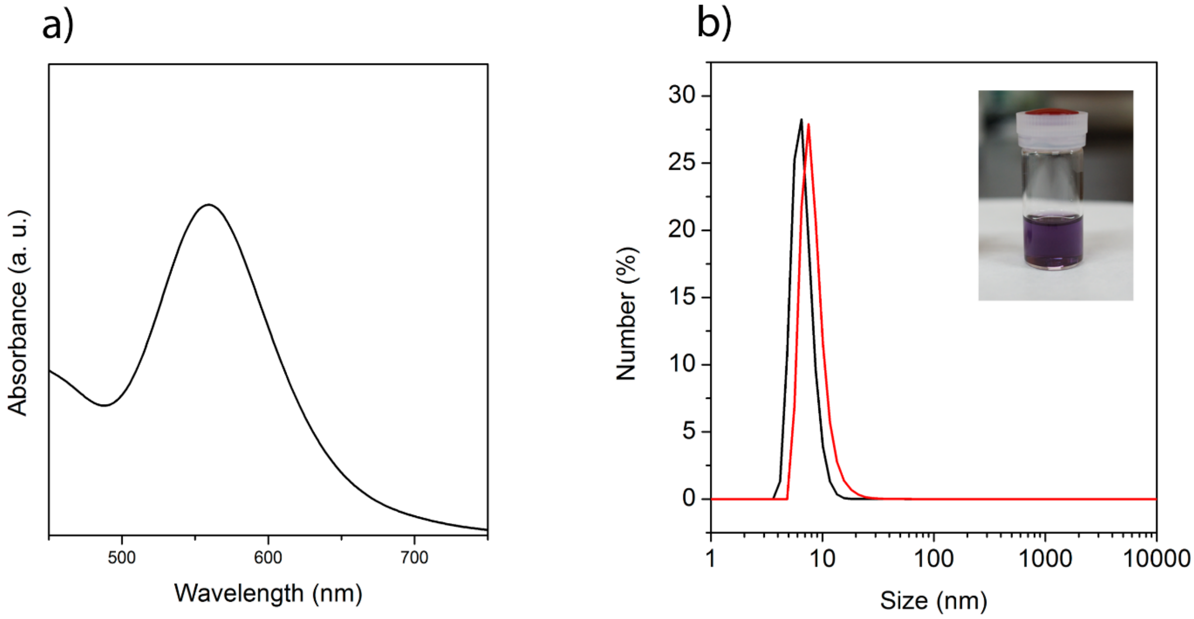

Supplementary Figure 18. (a) UV-Vis spectrum of the kinetically trapped molecule $C_{12}RhMOP(bix)_{12}$ (λ_{max} = 559 nm) (0.23 mM). (b) DLS measurements of the kinetically trapped $C_{12}RhMOP(bix)_{12}$ immediately after preparation (black) and after incubation at RT overnight (red). (Inset) A photograph of a DMF solution containing the kinetically trapped $C_{12}RhMOP(bix)_{12}$.

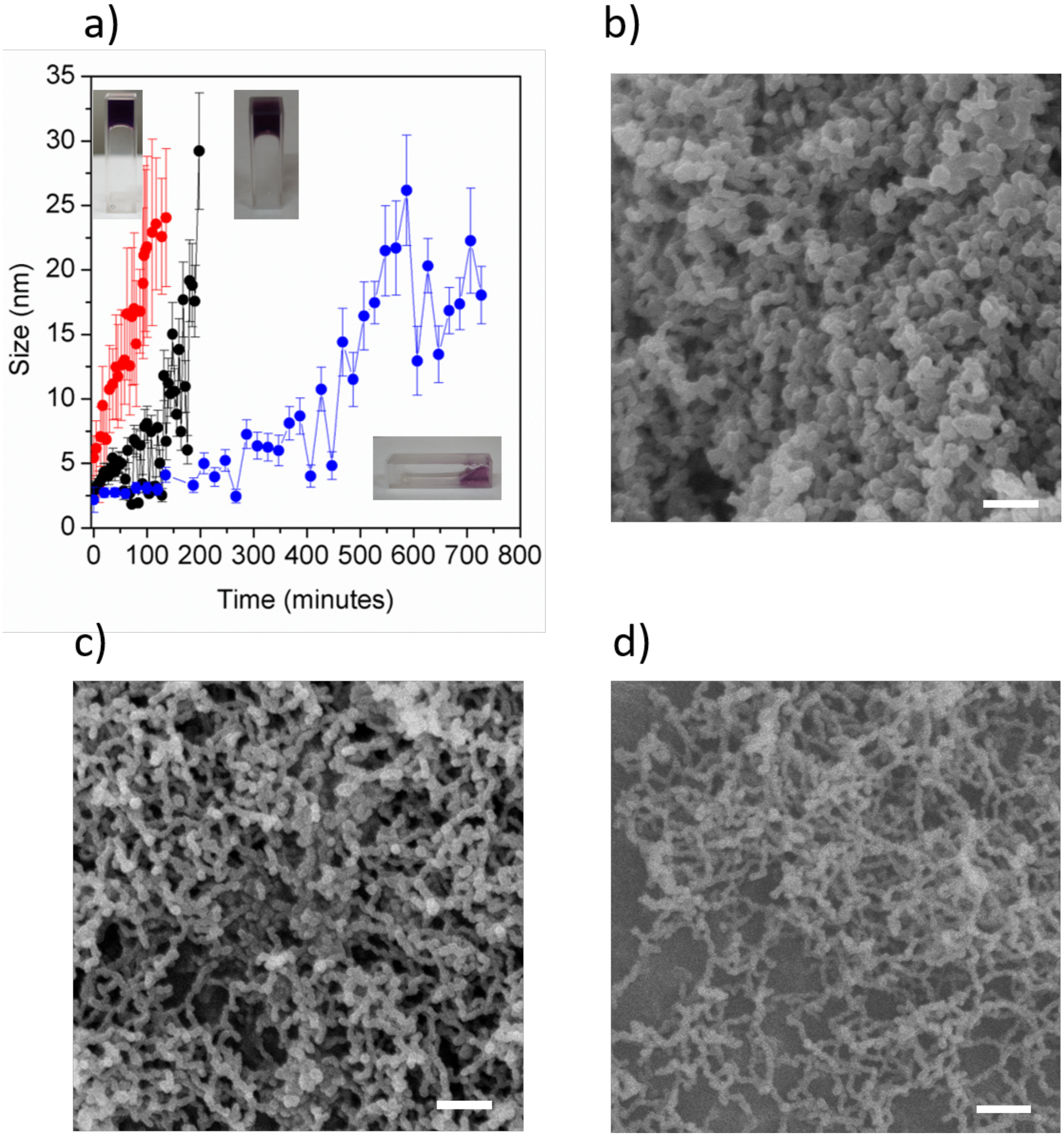

Supplementary Figure 19. Size evolution of the kinetically trapped $C_{12}RhMOP(bix)_{12}$ molecules when heated at 80 ºC, as followed with DLS measurements. MOP concentration was 1.83 mM (red), 0.93 mM (black) and 0.23 mM (blue). (Insets) Photos of the gels formed in the DLS cuvette during the measurements carried out at a concentration of 1.83mM (top left) and 0.93 mM (top center) and the weak gel formed at the concentration of 0.23 mM (bottom right). (b-d) Representative FESEM images of the aerogels obtained after the $SCCO_2$ drying of supramolecular gels synthesized with $C_{12}RhMOP(bix)_{12}$ at a concentration of 1.83 mM (b), 0.93 mM (c) and 0.23 mM (d). Scale bar: 200 nm. DLS monitoring and FESEM images reveal that the initial concentration of $C_{12}RhMOP(bix)_{12}$ impacts on the gelation time but not on the size of the fused colloids that compose the gel. However, at a higher concentration of the kinetically trapped precursors the coordination network is denser which allows the entrapment of the solvent.

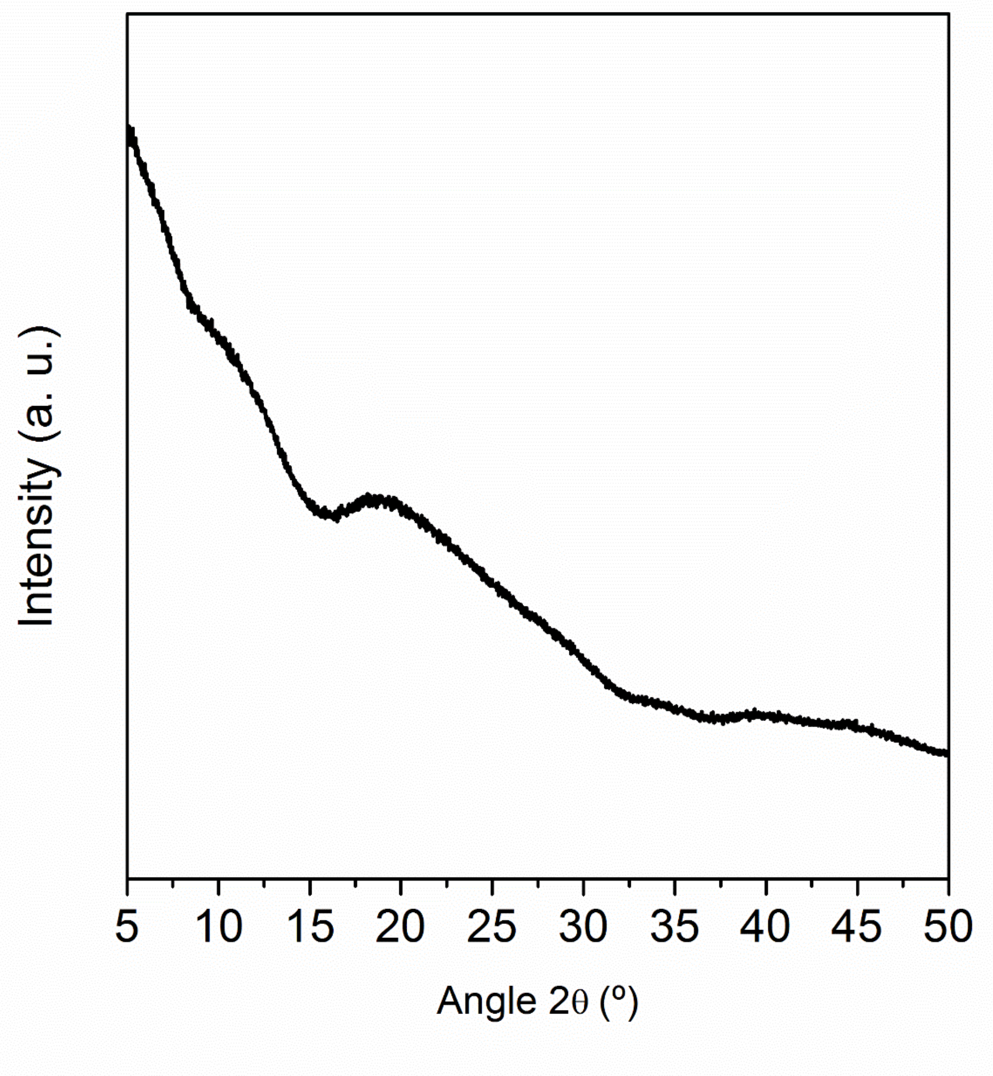

Supplementary Figure 20. Powder X-ray diffractogram of SCG-1.

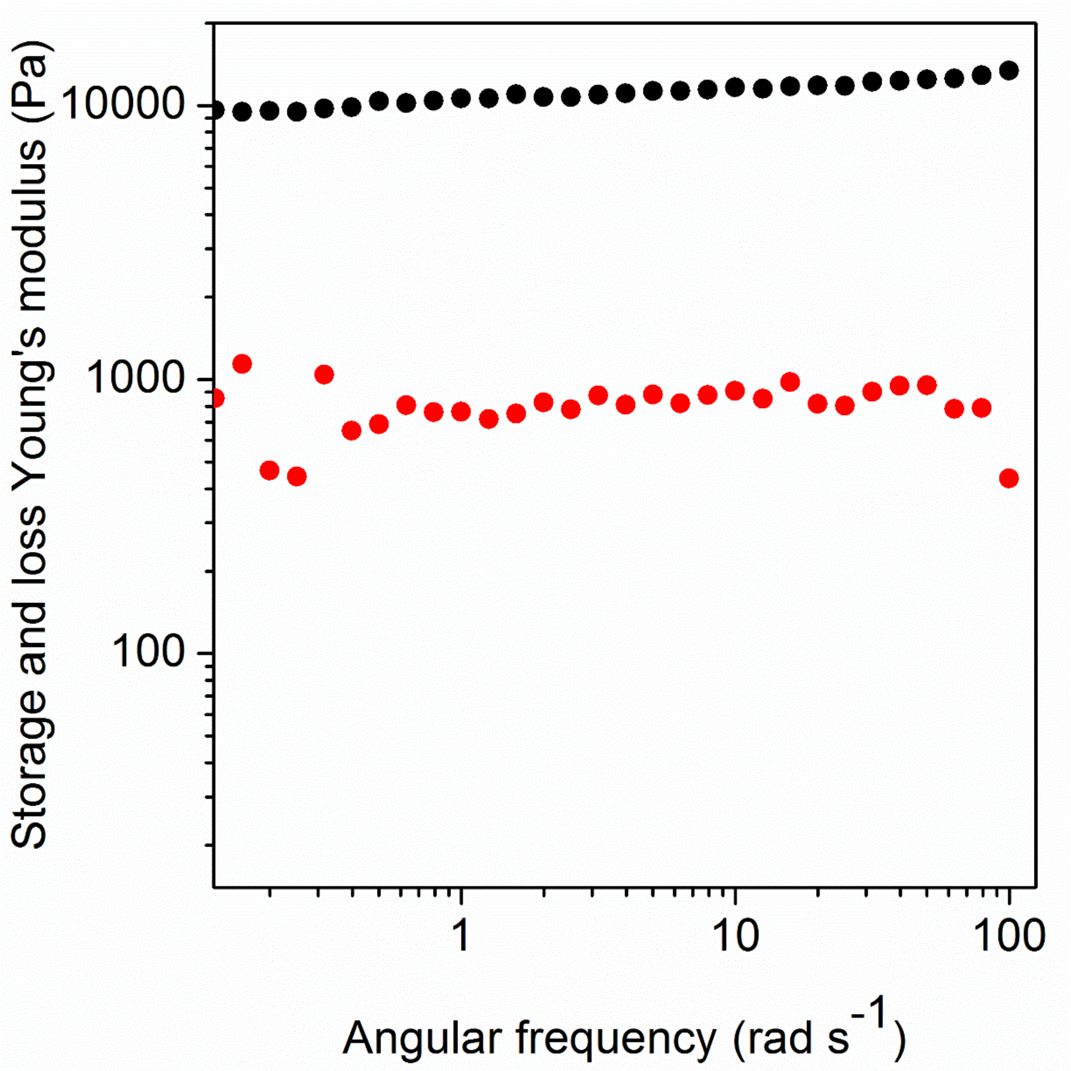

Supplementary Figure 21. Rheological measurements of SCG-1. Storage modulus (black) and loss modulus (red).

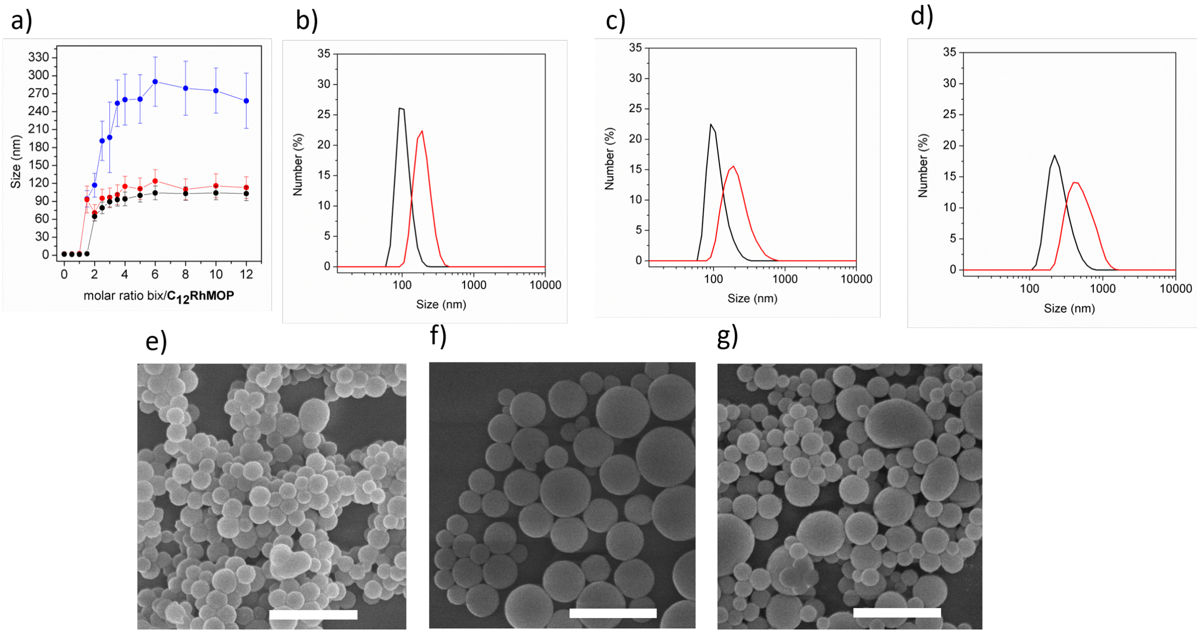

Supplementary Figure 22. (a) DLS titration at 80 ºC of C_{12}RhMOP with bix at a MOP concentration of 0.23 mM (black), 0.93 mM (red) and 1.8 mM (blue) (b-d) DLS measurements at the end of the titration (80 ºC) (black) and after cooling the suspension at RT (red). In each of the titrations, a size increase of the colloidal particles was observed upon cooling. This is explained by the fact that at high temperature and low concentration (at the end of the titration most of the reagents have reacted to yield CPPs) the coordination is inhibited. When the mixture is brought to RT the coordination is no longer inhibited and further growth on the surface of the particles becomes possible. (e-g) Representative FESEM images of the colloids obtained after the titration of C_{12}RhMOP with bix at 80 ºC at a MOP concentration of 0.23 mM (e), 0.93 mM (f), and 1.8 mM (g). Scale bar: 1µm. In all cases the chemical composition was found to be C_{12}RhMOP(bix)$_{7.9}$.

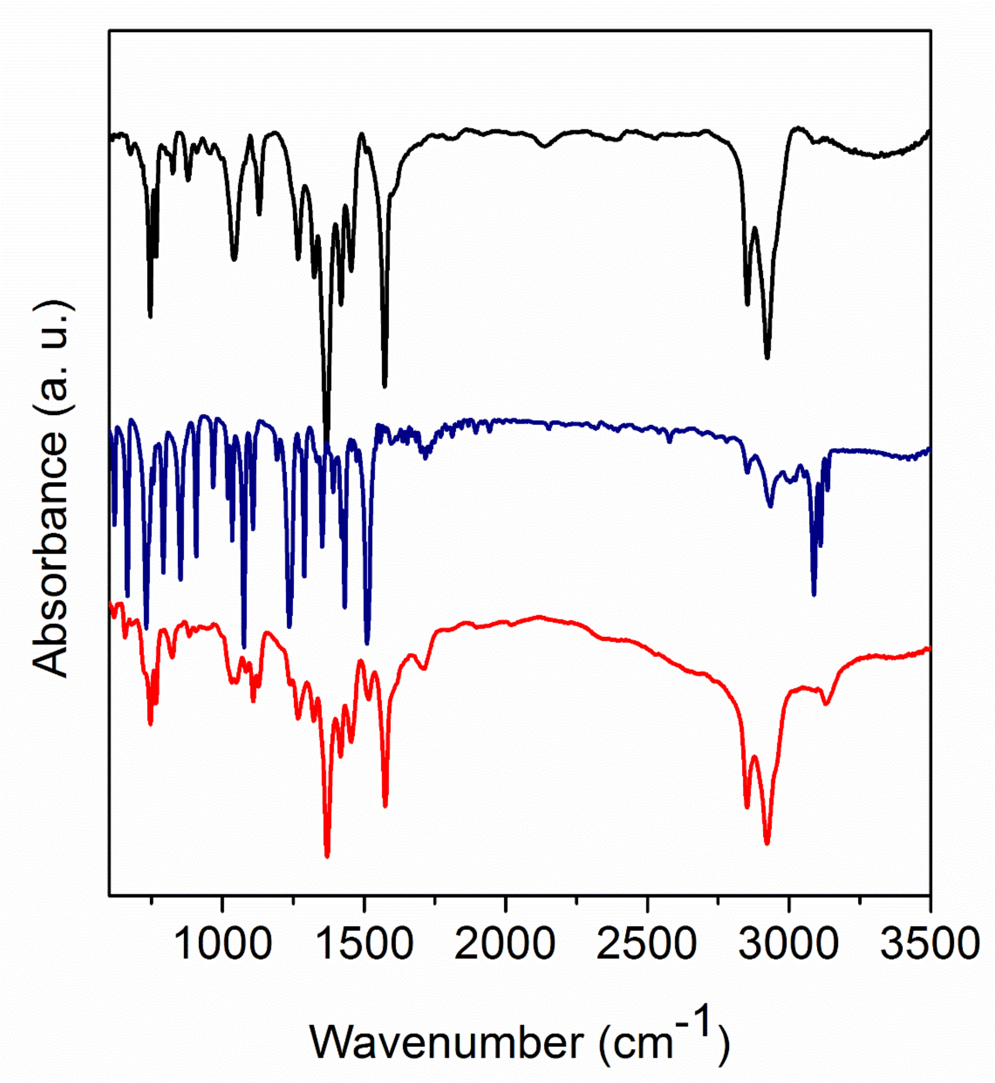

Supplementary Figure 23. FT-IR spectra of C_{12}RhMOP (black), bix (blue), and SCG-1 (red).

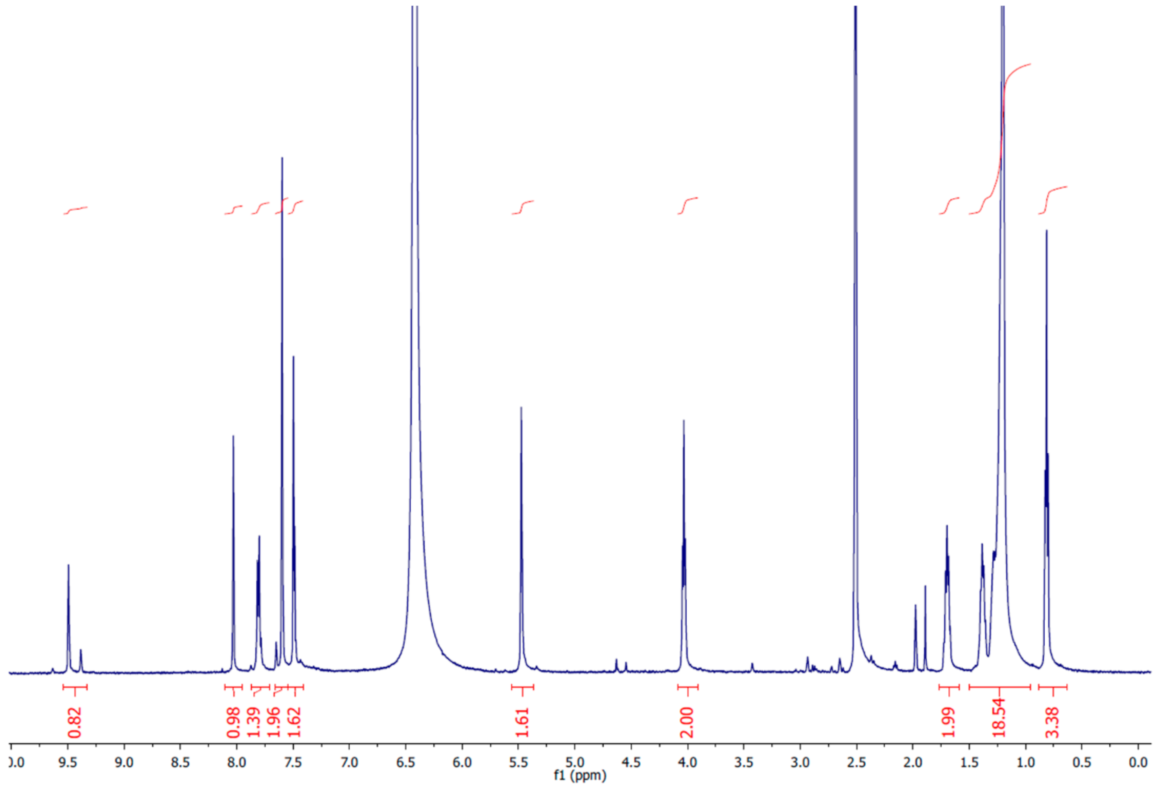

Supplementary Figure 24. ^{1}H-NMR spectra of digested SCG-1. The corresponding formula was C_{12}RhMOP(bix)$_{9.7}$.

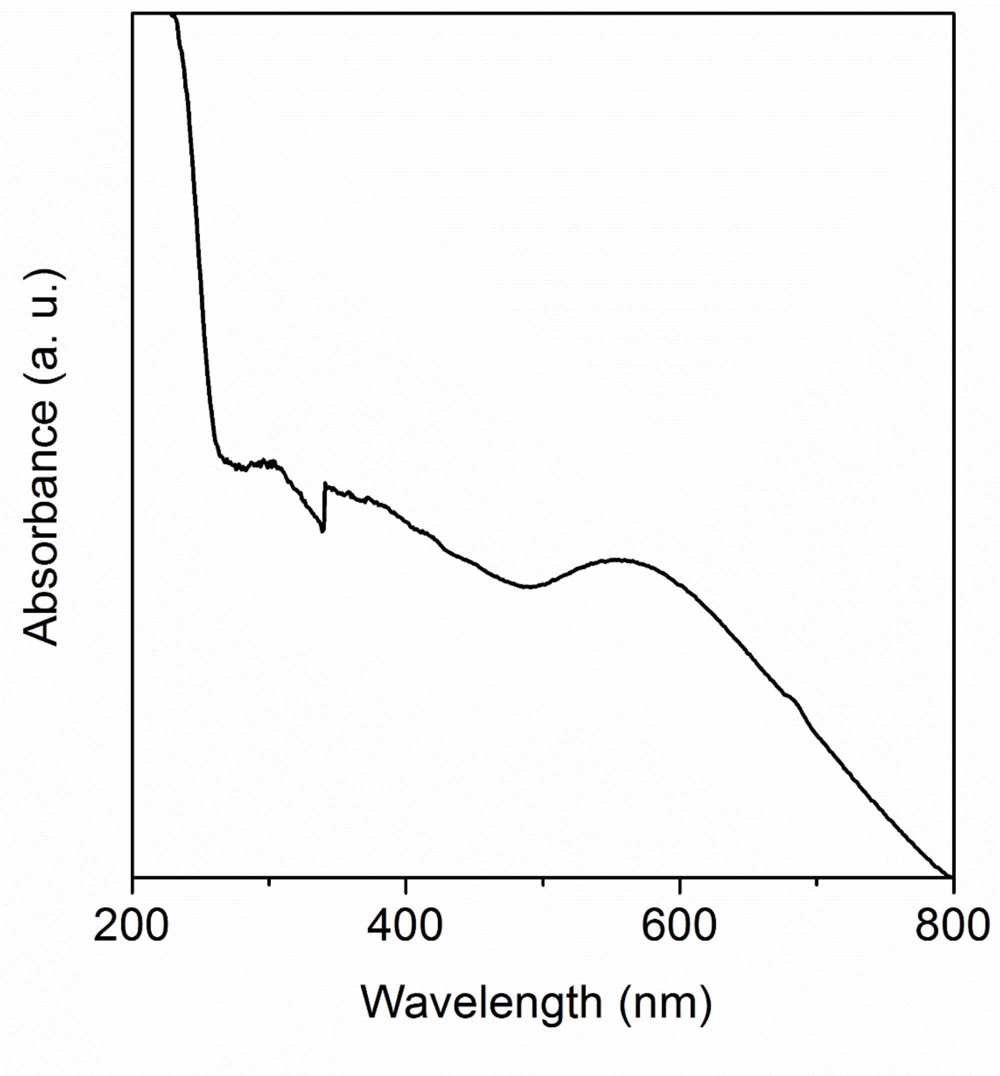

Supplementary Figure 25. Solid state UV-Vis spectrum of SCG-1; λ_{max} = 560 nm.

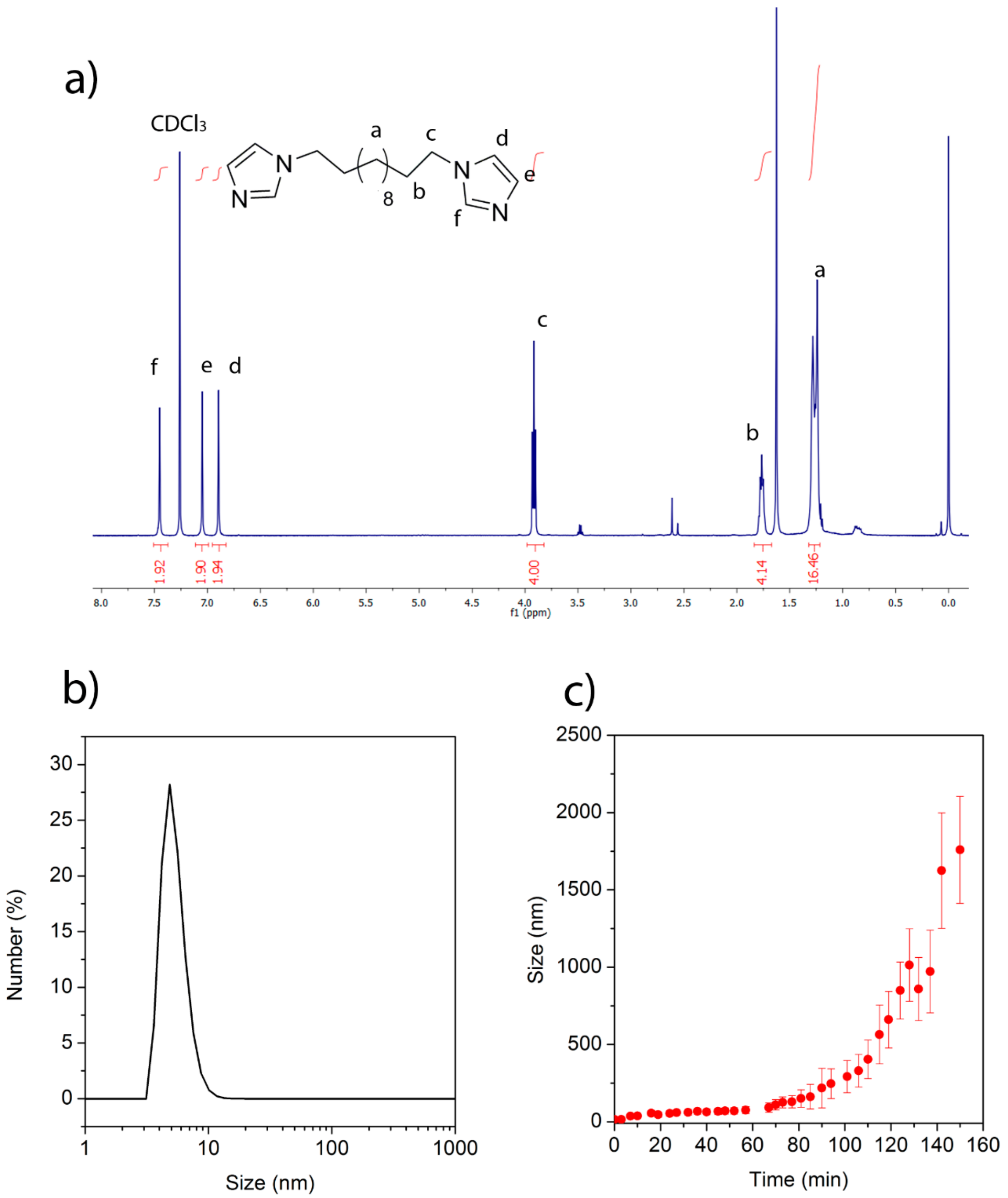

Supplementary Figure 26. (a) ^{1}H-NMR spectrum of bidod. (b) DLS measurement of the kinetically trapped species after reacting C_{12}RhMOP (1.83 mM) with 12 mol. eq. of bidod. c) Size evolution of the kinetically trapped molecule when heated at 80 ºC, followed using DLS.

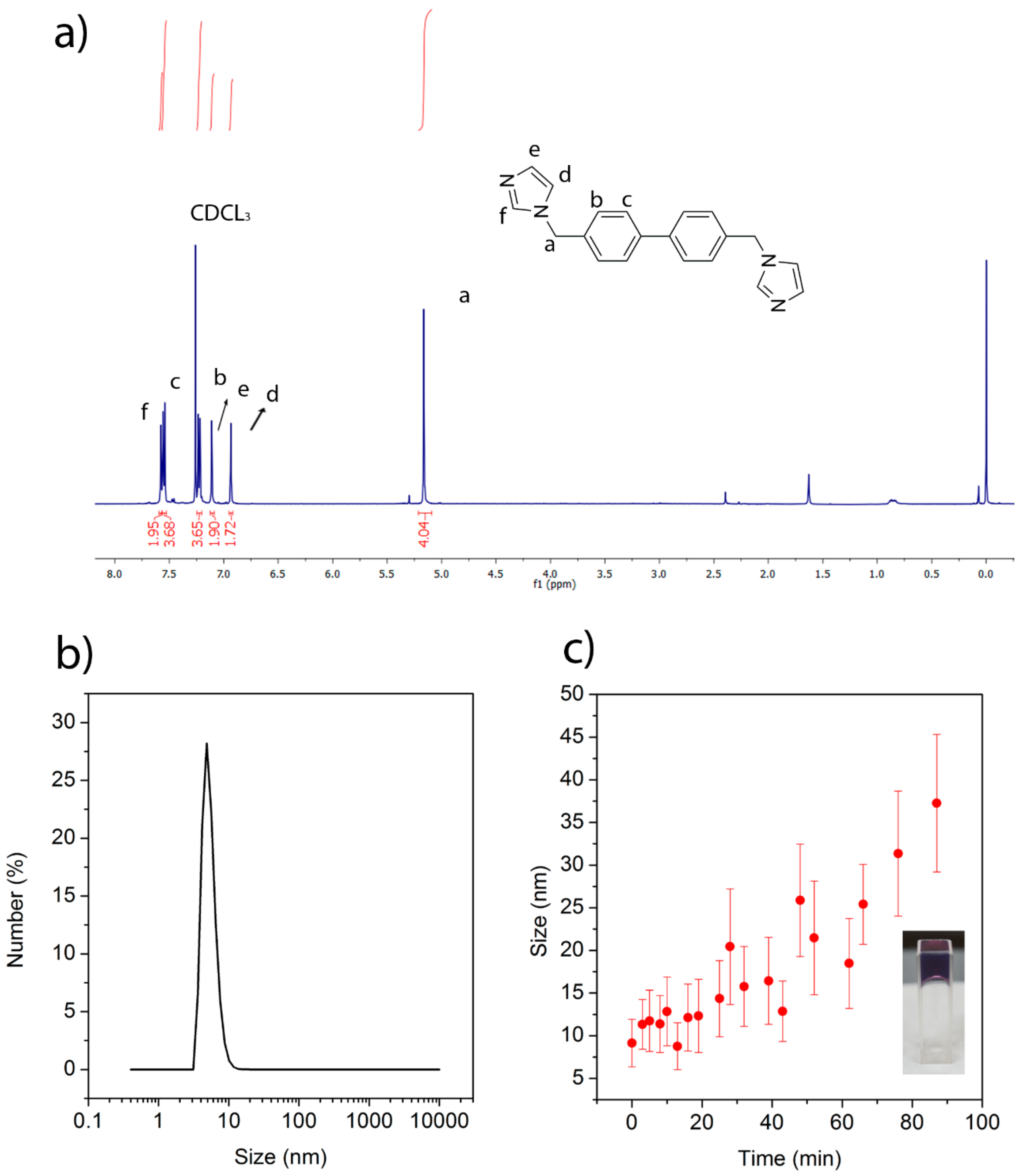

Supplementary Figure 27. (a) ^{1}H-NMR spectrum of bibPh. (b) DLS measurement of the kinetically trapped species generated when C_{12}RhMOP (1.83 mM) is reacted with 12 mol. eq. of bibPh. (c) Size evolution of the kinetically trapped molecule when heated at 80 ºC, followed using DLS. (Inset) Photograph of the synthesized gel.

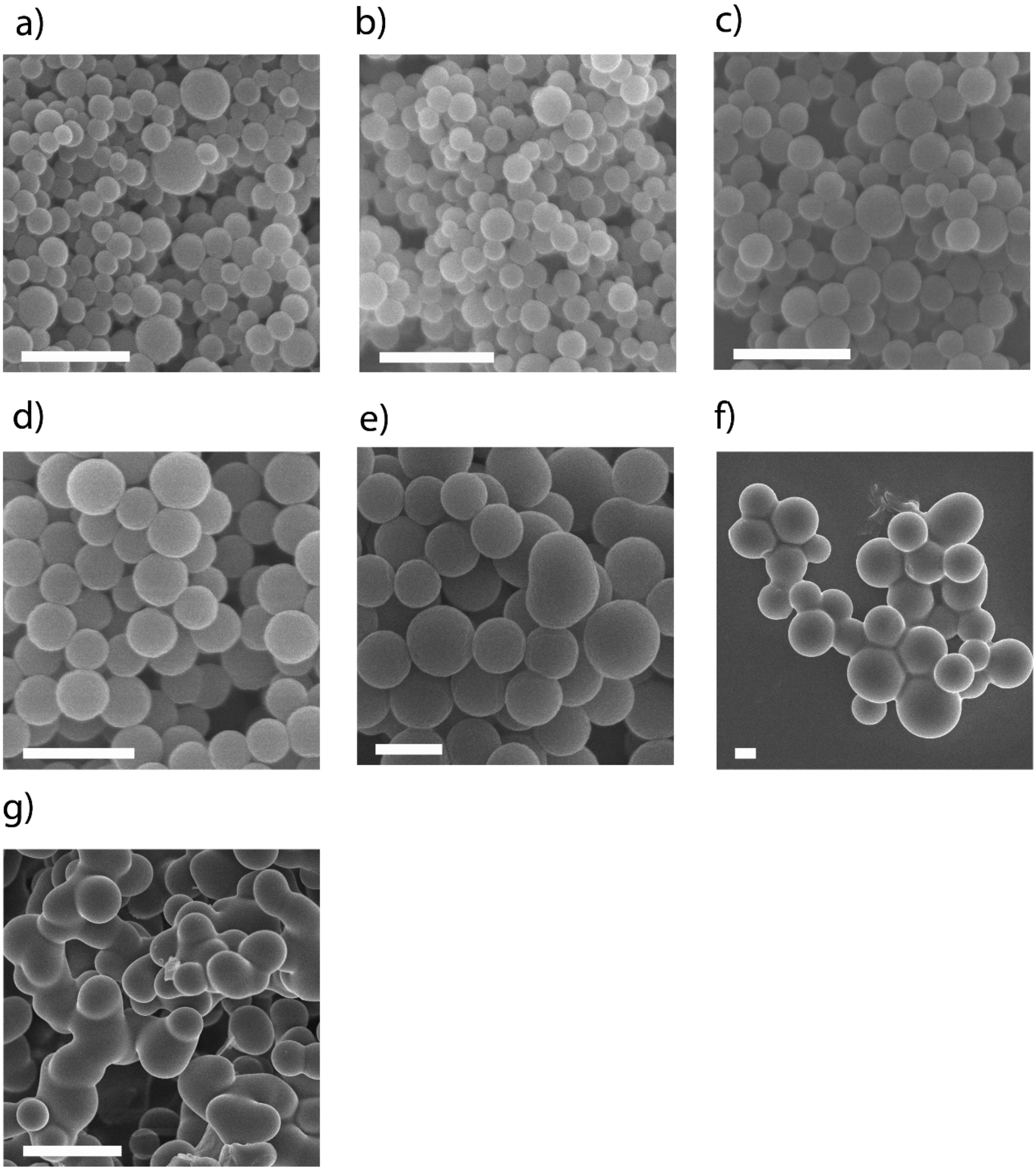

Supplementary Figure 28. Evolution of the reaction of C_{12}RhMOP with bidod, as monitored by FESEM. From (a) to (g) the images correspond to the following reaction times: 20 min, 60 min, 80min, 120min, 150min, 180 min and overnight. The mechanism is analogous to that described in the main text for SCG-1 (i. e. colloidal particle formation and subsequent fusion of these particles). However, in this case the initial colloids are bigger, which causes phase segregation when the fusion stage begins, preventing gel formation. Instead, fused microparticles are produced (FMP-1). Scale bars: scale bar (a-f) = 500 nm and scale bar (g) = 5 μm.

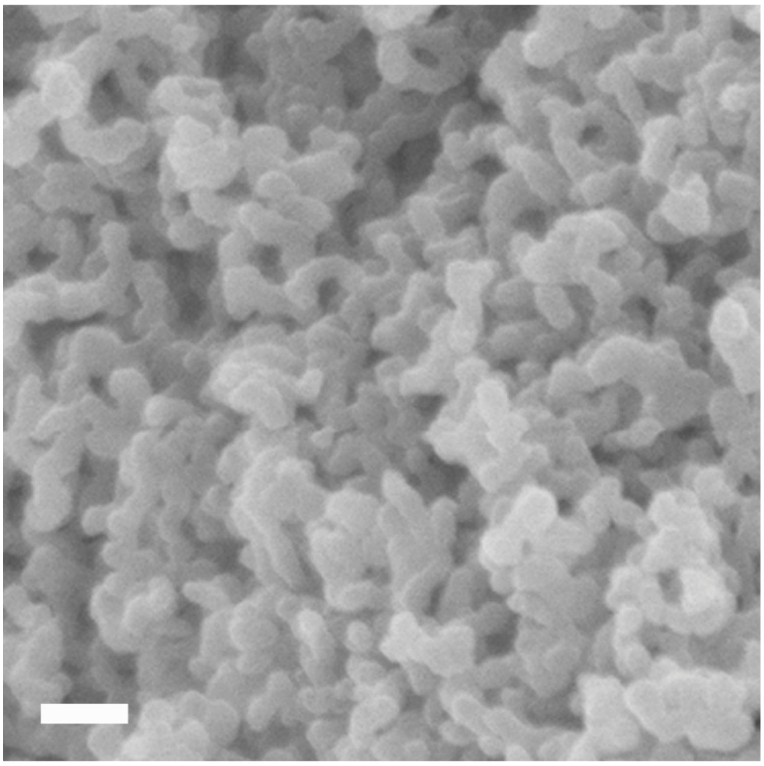

Supplementary Figure 29. FESEM image of the aerogel derived from the reaction of C_{12}RhMOP with bibPh; scale bar = 100 nm.

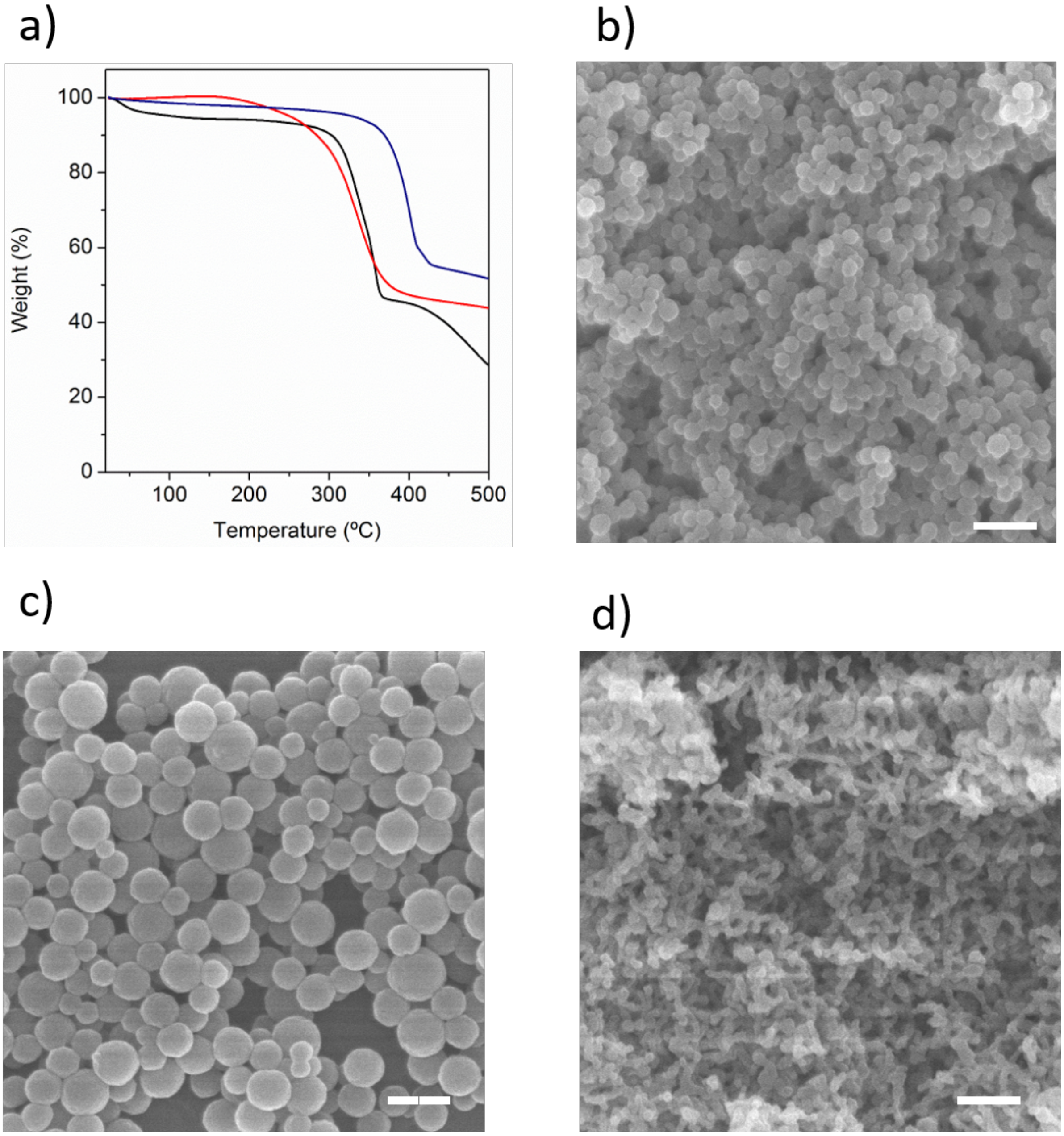

Supplementary Figure 30. (a) TGA measurements for C_{12}RhMOP (black), CPP-1 (blue) and SAG-1 (red) confirm their thermal stability at the conditions used for their activation. (b-d) FESEM images of CPP-1_small (b), CPP-1_big (c) and SAG-1 after the activation process. In all the cases the activation process does not modify the morphology of the initial material. Scale bars: 200 nm.

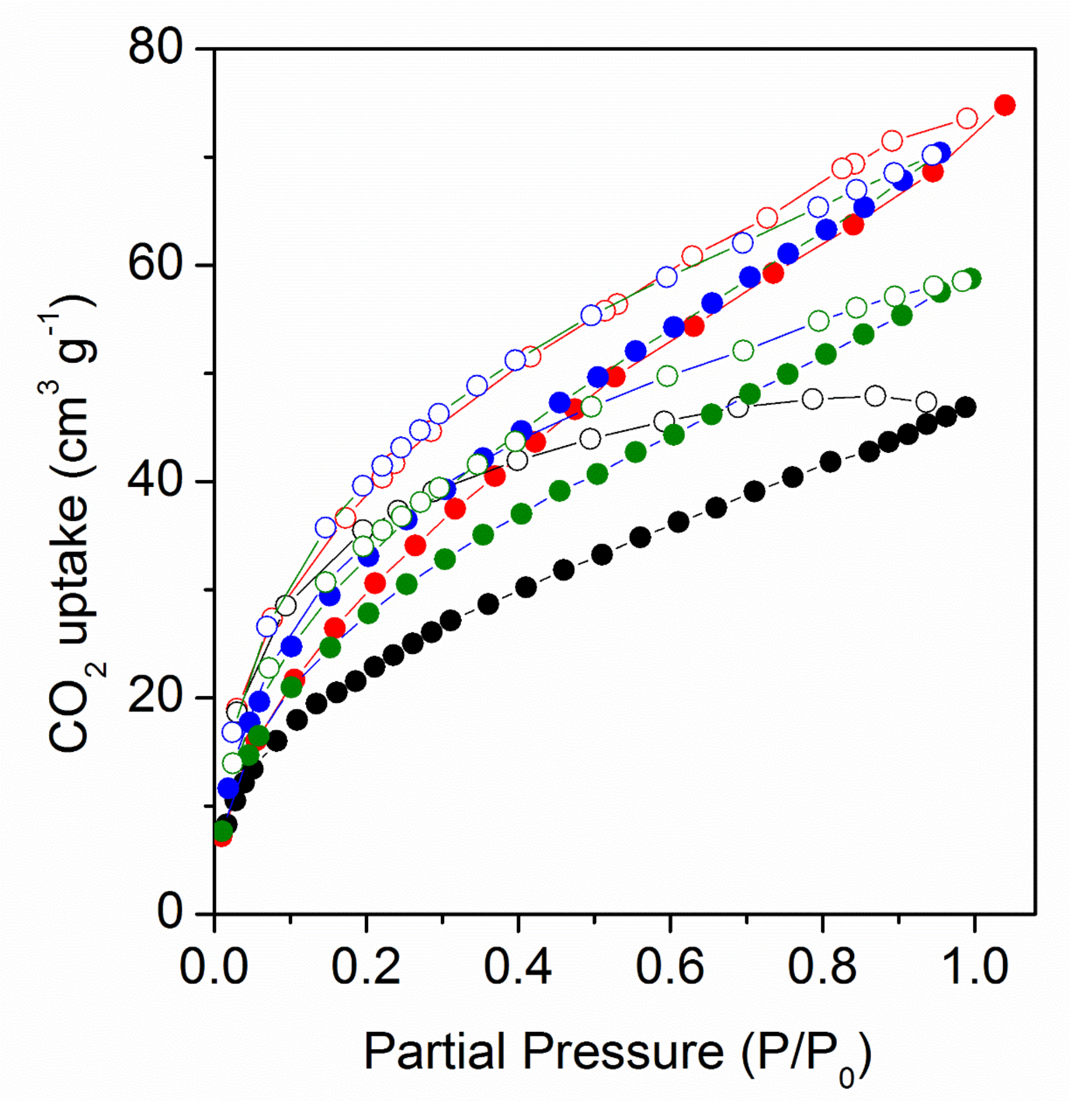

Supplementary Figure 31. CO_2 uptake at 195 K of SAG-1 (red), CPP-1_small (blue), CPP-1_big (green) and C_{12}RhMOP (black). Filled symbols depict adsorption and empty symbols, desorption.

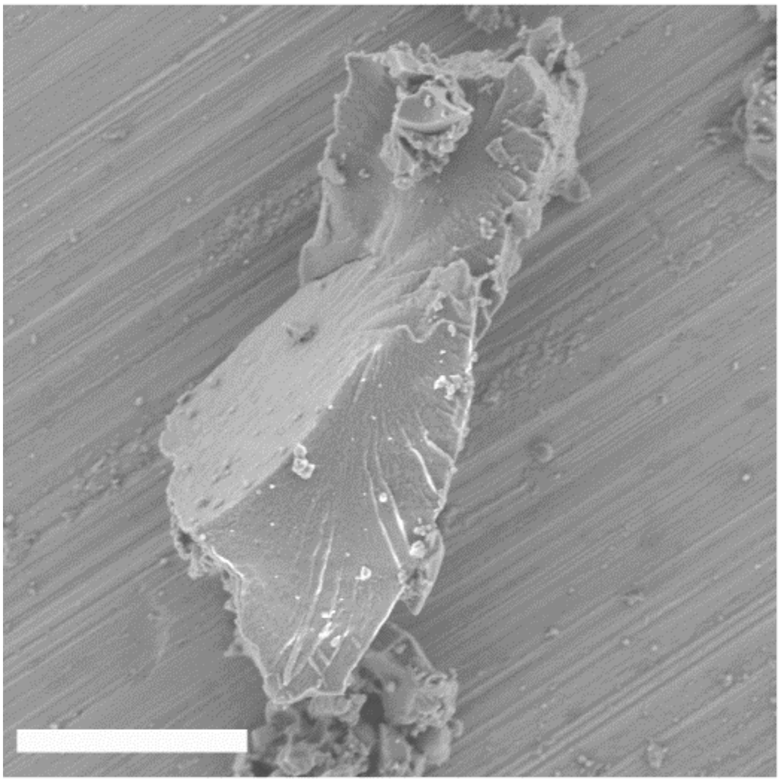

Supplementary Figure 32. Representative FESEM image of C_{12}RhMOP. Scale bar: 5 μm.

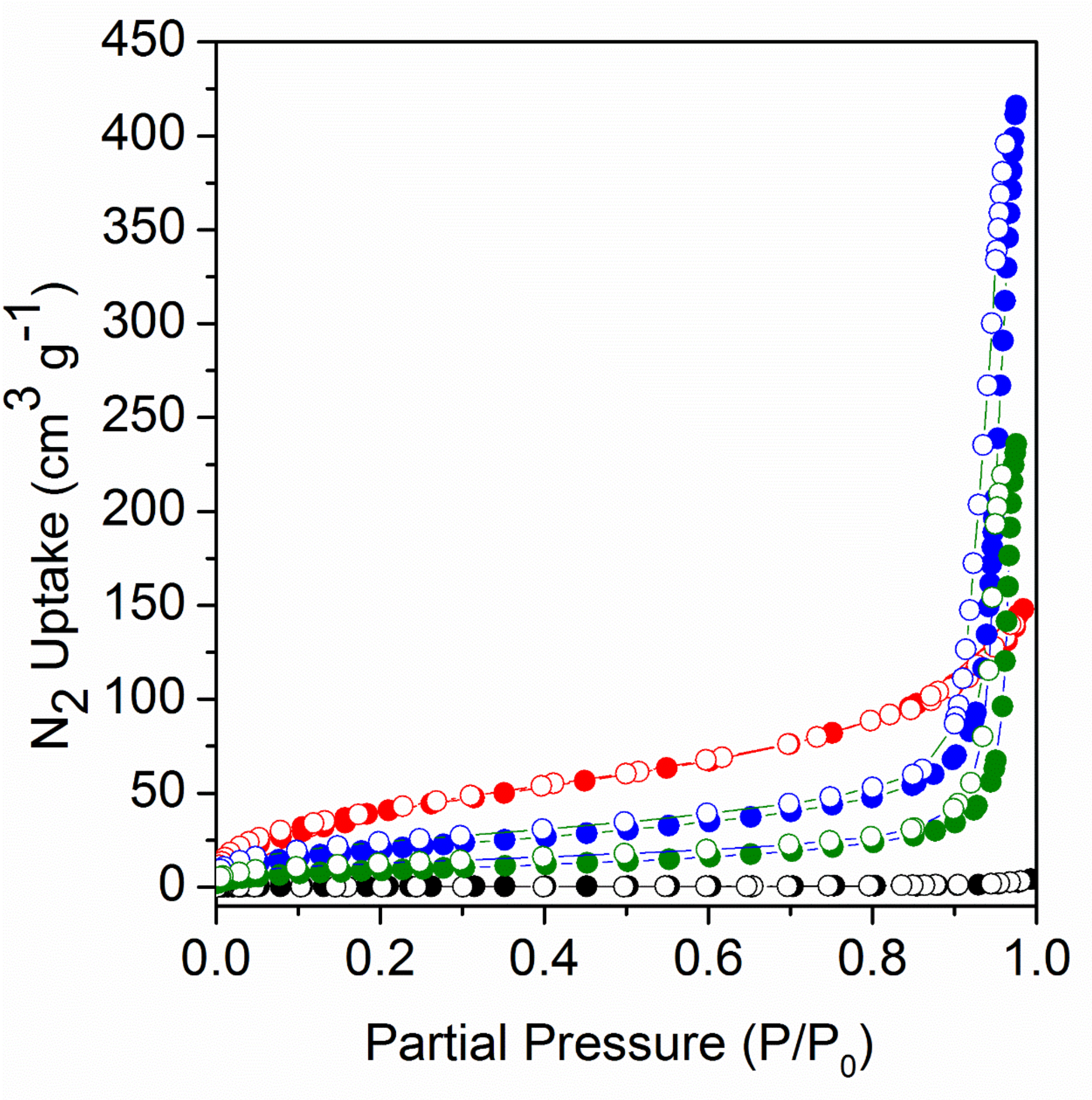

Supplementary Figure 33. N_2 uptake at 77 K of SAG-1 (red), CPP-1_small (blue), CPP-1_big (green) and C_{12}RhMOP (black). Filled symbols depict adsorption and empty symbols, desorption.

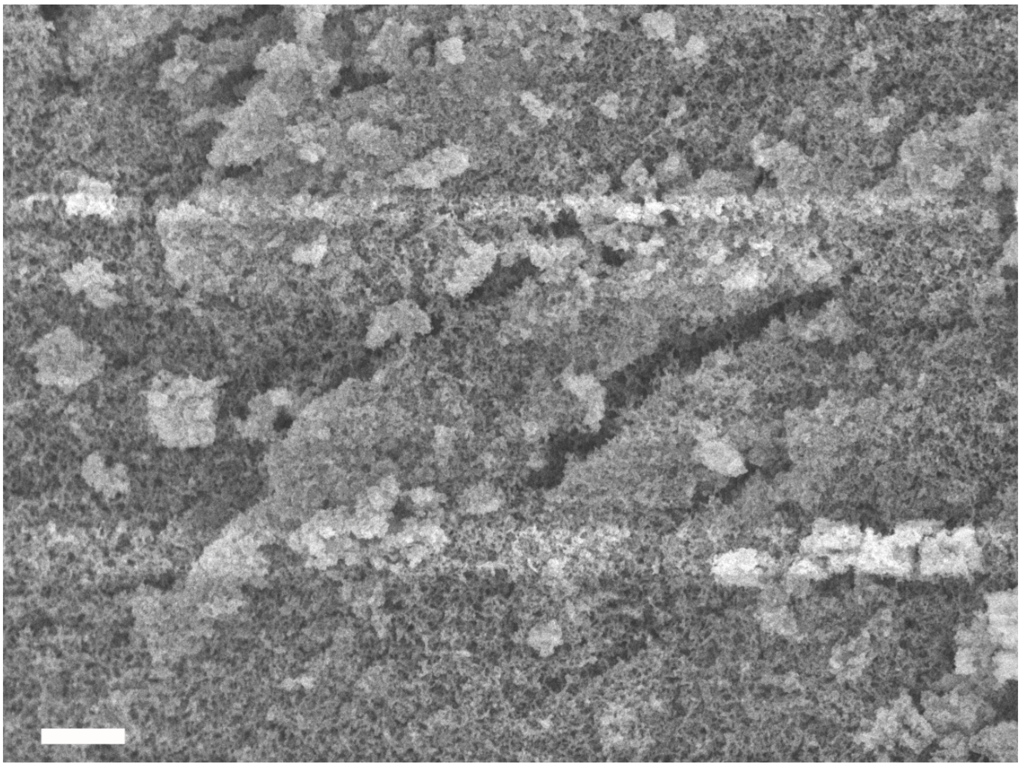

Supplementary Figure 34. Low magnification FESEM image of SAG-1 showing its hierarchical porous structure; scale bar = 1 μm.

Supplementary Methods

Instrumentation

All reagents and reactants were purchased from Wako Pure Chemical Industries and were used without further purification. PXRD measurements were performed using a Rigaku Smartlab (Dtex Ultra detector) operating with a rotating anode Cu Kα X-ray generator (λ = 1.54 Å) with a 40 kV beam voltage and 200 mA current. Thermogravimetric analyses (TGA) were carried out in the temperature range from room temperature to 500 ºC at a heating rate of 10 C min^{-1}, with a Rigaku Thermo plus EVO2, under nitrogen atmosphere. The super-critical CO_2 drying process was carried out on SCLEAD-2BD autoclave (KISCO) using super-critical CO_2 at 14 MPa and 40 ºC. The gas sorption isotherms were recorded on a BELSORP-max volumetric adsorption instrument from BEL Japan, Inc.. Infrared (IR) spectroscopy data were recorded using a Jasco FT/IR-6100 equipped using KBr pellets with a 1 cm^{-1} resolution and an accumulation of 64 scans. Samples were observed using a field-emission scanning electron microscope with a JEOL Model JSM-7001F4 system operating at 5 kV and 5 mA current. Linear dynamic mechanical analysis was performed using RSA-G2 (TA instruments) by compression mode. ^{1}H-NMR spectra were recorded on a Bruker Biospin DRX-600 (600 MHz) spectrometer. Dynamic Light Scattering measurements were performed using a Zetasizer Nano ZS.

Synthesis of ligands

Synthesis of 1,4-bis(imidazol-1-ylmethyl)benzene (bix)

The synthesis was adapted from reported procedures.[1] In a typical synthesis, imidazole (2.13 g, 16.6 mmol) and NaH (0.88g of 60% in mineral oil) were mixed at RT in THF (50 ml) for 30 min. Then, a solution of para-bromo xylene (2 g, 7.57 mmol) in 20 ml of THF was added and

the temperature was increased to 50 ºC for 4h. The reaction was quenched by adding ice water (25 ml) and the organic phase extracted with chloroform, treated with anhydrous $MgSO_4$ and dried under vacuum. The white powder was washed with diethyl ether twice and finally dried under vacuum.

Synthesis of 4,4'-Bis(imidazol-l-ylmethyl)biphenyl (bibPh).

The synthesis was adapted from reported procedures in the same manner as described for bix.[1]

Synthesis of 1-dodecyl-1H-imidazole (dis)

The synthesis of dis was adapted from reported procedures.[2] 1.63 g of imidazole (24 mmol) and 4.97 g of K_2CO_3 were mixed in acetonitrile (60 ml) and stirred at RT for 2 h. Then 1-bromododecane (4.98 g, 20 mmol) is added and the solution was stirred for 24 h at RT. Finally, the solvent was evaporated and water (35 ml) was added to the resulting solid. The product was extracted in DCM (40 ml × 3), treated with anhydrous $MgSO_4$, filtered, and dried under vacuum.

Synthesis of 1,1'-(1,12-Dodecanediyl)bis[1H-imidazole] (bidod)

The synthesis was performed according to reported procedures.[3]

Sample preparation for rheological measurements

Gelation was performed in a syringe. After the reaction, the gel was removed from the syringe and kept in 3,5,5-Trimethyl-1-hexanol in order to prevent solvent evaporation during rheological measurements.

Sample digestion

5-10 mg of the sample were dispersed in a mixture of DMSO-d^6 (750 µl) and DCl (20 µl). The mixture was heated at 100 ºC overnight to obtain a yellow solution.

Supplementary References

1. P. K. Dhal, F. H. Arnold, *Macromolecules*, 1992, **25**, 7051-7059

2. L. Casal-Dujat, Ma. Rodrigues, A. Yagüe, A. C. Calpena, D. B. Amabilino, J. González-Linares, M. Borràs, L. Pérez-García, *Langmuir*, 2012, **28**, 2368-2381.

3. J.-F. Xu, Y.-Z. Chen, L.-Z. Wu, C.-H. Tung, Q.-Z. Yang, *Org. Lett.* 2013, **15**, 6148-6151.
